# Relative reinforcer efficacy and persistence in extinction are significantly correlated but not clinically actionable

**DOI:** 10.1002/jaba.70097

**Published:** 2026-11-08

**Authors:** Joseph M. Lambert, Allyson L. Hayward, Caitlin Price, Jessica A. R. Logan

**Affiliations:** ^1^ Department of Special Education Vanderbilt University Nashville TN USA

**Keywords:** behavioral economics, challenging behavior, extinction bursts, functional analysis, response persistence

## Abstract

Although assessments of relative reinforcer efficacy (RRE) have demonstrated utility for instructional programming, their relevance to informing treatment for challenging behavior is underexplored. Because reinforcer value moderates response persistence, RRE may serve as an index of persistence and could have prescriptive utility for treatment design. We targeted the persistence of arbitrary responses of 90 consenting and compensated adults with disabilities. We assigned participants to one of five baselines in which target responses were maintained on schedules prescribed by demand curve analysis (i.e., elastic‐EO, inelastic‐EO, elastic‐AO, inelastic‐AO, *P*_*max*_) using reinforcers whose RRE had been quantified by breakpoints obtained during progressive‐ratio analysis. We then placed responding on extinction. Linear regressions revealed significant correlations between breakpoints and various metrics of persistence (i.e., rate, latency, reinforcers missed). These relations, however, were less apparent via visual inspection. Our data suggest progressive‐ratio analysis will not serve clinically useful functions without additional conceptual and validation work.

There are multiple evidence‐based options for addressing challenging behavior (e.g., antecedent‐based strategies, differential reinforcement; Steinbrenner et al., 2020). Although some data, and a general consensus among clinicians, suggest that the ideal approach to intervention design entails focusing on the function(s) of challenging behavior (Jeong & Copeland, 2020; Lambert & Houchins‐Juárez, 2020; Melanson & Fahmie, 2023; Muharib & Gregori, 2022), there is little research to indicate which function‐based intervention options are likely to be the most effective or appropriate for a given case (Lambert, Copeland, et al., 2022; Lambert, Paranczak, et al., 2022; Lambert, Sandstrom, et al., 2022).

For example, a breadth of evidence demonstrates that operant extinction can produce rapid reductions in challenging behavior (Fisher et al., 2018, 2019; Iwata et al., 1994; Miller et al., 2022). Notwithstanding, its use in treatments for challenging behavior has been repeatedly associated with undesirable collateral effects (e.g., extinction bursts), which are untenable in some applied settings (Ducharme & van Houten, 1994; Lerman & Iwata, 1995; Lerman et al., 1999; MacNaul & Neely, 2018). Currently, little is known about why many of the adverse collateral effects of extinction, like extinction bursts, occur (Fisher et al., 2023; Lerman & Iwata, 1996; Shahan, 2022). Existing evidence suggests that the magnitude of extinction bursts and persistence of behavior in extinction more broadly are moderated by the relative density of baseline reinforcement schedules (Nevin & Grace, 2000) as well as the presence (or lack thereof) of competing alternative reinforcer options during extinction (Nist & Shahan, 2021; e.g., Shukla & Albin, 1996). Perhaps as a consequence, the procedure is rarely used in isolation in clinical practice and is more often paired with alternative reinforcement (e.g., Hagopian et al., 2004).

Recently, Lambert et al. (2024) also demonstrated that baseline unit prices can interact with fluctuations in motivating operations (Laraway et al., 2003) to both exacerbate and mitigate persistence and bursting. Specifically, Lambert et al. (2020) randomly assigned 69 adults with disabilities to one of four experimental conditions in a translational investigation targeting arbitrary responses to explore whether it was possible to influence responding during extinction by scaling and manipulating baseline reinforcement parameters in relation to the behavioral economic principles of equilibrium and *P*_*max*_ (Hursh, 1980, 1984).

Equilibrium describes a “sweet spot” in terms of preferred levels of responding and reinforcer consumption that balances contextual factors including reinforcer availability and other relevant selection pressures (e.g., the “openness” of the reinforcer economy in terms of reinforcers available previously or elsewhere outside of session; Hursh, 1980, 1984). In behavioral economics research, equilibrium is typically quantified by sampling response outputs and reinforcer consumption in experimental arrangements that do not restrict how many reinforcers participants can earn and consume (Lambert et al., 2026). Importantly, equilibrium is context dependent and generally decreases as unit prices are increased (Gilroy et al., 2020; Reed et al., 2025).

The construct of demand describes how equilibrium's component variables (i.e., response output and reinforcer consumption) vary as a function of changes in unit price. Specifically, when increases in unit price lead to increased response output, demand is *inelastic*. When increases in unit price lead to decreased response output, demand is *elastic* (Madden et al., 2024). Demand curve analysis is a parametric analysis concerned with quantifying how the slopes of equilibrium (i.e., responding and consumption) change as a function of economic constraint. The objective of demand curve analysis is to establish a commodity's *essential value* (Gilroy, 2023)—the degree to which consumption is affected by price, with higher essential value meaning consumption is less affected by increases in price. Although *P*_*max*_ (the unit price that produces the maximum response output, or *O*_*max*_) is often the focus of demand curve analysis because it identifies a given reinforcer's optimal price point (e.g., Gilroy et al., 2025), metrics of equilibrium—demand intensity (i.e., net reinforcer consumption when access is unconstrained) and *O*_*max*_—are also produced by the analysis and offer useful information that can be directly relevant to intervention design (e.g., Murphy et al., 2025).

Relevant to extinction, Lambert et al. (2024) hypothesized that bursts and persistence were the product of an interaction between at least two important moderators that could be precisely scaled by demand curve analysis—the position of baseline unit prices relative to *P*_*max*_ (i.e., prior to extinction, were participants “underpaying” or “overpaying” for programmed reinforcers?) and the position of reinforcer consumption patterns relative to demand intensity (i.e., prior to extinction, how close were participants to “meeting their needs” with respect to programmed reinforcers?).

Using blocked randomization, Lambert et al. (2024) enrolled participants over a 2‐year period and matched participant tetrads according to similarities in breakpoints produced by targeted reinforcers during progressive‐ratio analysis (PRA). Through a subsequently conducted demand curve analysis, the authors identified reinforcer‐specific *P*_*max*_ values for each participant (i.e., unit prices that yielded the greatest net response outputs) and randomly assigned participants to one of four groups—inelastic establishing operation (EO), elastic EO, inelastic abolishing operation (AO), and elastic AO. Participants assigned to inelastic groups were then exposed to a baseline condition in which response requirements were lower than *P*_*max*_ (i.e., rich schedules of reinforcement). Participants assigned to elastic groups were exposed to a baseline condition in which response requirements were higher than *P*_*max*_ (i.e., lean schedules of reinforcement). Immediately prior to extinction, participants assigned to AO groups were allowed to consume one less than the total number of reinforcers, which defined demand intensity (i.e., the equilibrium between supply and demand when programmed reinforcers were freely available; see also *diminishing marginal utility* in Madden et al., 2024). Alternatively, participants randomly assigned to EO groups only consumed a single reinforcer prior to extinction onset.

During extinction, the responding of participants who had been deprived of reinforcement (i.e., EO) was more likely to persist than the responding of participants whose preextinction consumption had approximated equilibrium (i.e., AO). This effect was amplified when unit prices were on the elastic portion of the demand curve (i.e., when schedules of reinforcement were relatively thin), leading the authors to conclude that the costs of persistence‐mitigation techniques informed by behavioral momentum theory (e.g., waiting to implement extinction until baseline reinforcement rates have first been thinned; e.g., Lambert et al., 2016) may outweigh their benefits.

However, despite statistically significant main and interaction effects across experimental conditions, no single experimental manipulation in Lambert et al. (2024) completely circumvented extinction bursts—suggesting that persistence in extinction was moderated by other variables in addition to unit price and fluctuations in MOs resulting from preextinction consumption patterns. Whereas multiple unconsidered variables likely influenced persistence in this data set, one possible culprit was reinforcer value.

For context, some researchers have proposed that the response output supported by a given reinforcer is the best and most appropriate measure of its value (e.g., Francisco et al., 2008; Hodos, 1961). However, response outputs supported by a reinforcer when response requirements are low are not always predictive of responding when requirements are increased (Tustin, 1994). Consequently, systematic preference assessments and reinforcer analyses that use fixed‐ratio (FR) 1 schedules can be poor predictors of reinforcer efficacy when contingency parameters are increased to mirror those in typical contexts (e.g., DeLeon et al., 1997; Francisco et al., 2008; Penrod et al., 2008).

In this respect, PRA may be an underappreciated assessment methodology with implications for informing behavioral treatment. Broadly, PRA can be conceptualized as an analysis of diminishing returns that exposes participants to a sort of “hyperinflation” that documents the moment at which a targeted reinforcer is no longer worth the increasing effort required to obtain it. More specifically, PRA is an assessment in which participants are exposed to rapid and systematic schedule thinning. Unlike demand curve analysis, all schedule thinning during PRA occurs within a single session and occurs contingent on reinforcer delivery and consumption (e.g., Hodos, 1961; Jarmolowicz & Lattal, 2010). During the assessment, researchers often note the last schedule value for which responding yielded at least one programmed reinforcer (i.e., its breakpoint [BP1]). Then, they compare a reinforcer's breakpoint to the breakpoints of other reinforcers to establish its relative reinforcer efficacy (RRE) in ways analogous to the rankings generated by preference assessments (Gilroy et al., 2018, 2019; Hodos, 1961; Johnson & Bickel, 2006; Lambert et al., 2019; Madden et al., 2007a, 2007b; Reed et al., 2009; Rodefer & Carroll, 1997; Tustin, 1994).

In the applied literature, RRE has been used to predict the influence of contingent access to social interactions (Jerome & Sturmey, 2008, 2014), low‐preference stimuli (Francisco et al., 2008; Glover et al., 2008; Penrod et al., 2008), and various quantities of a given type of reinforcement (e.g., Trosclair‐Lasserre et al., 2008). It has also been used to inform reinforcer selection for the treatment of challenging behavior (Roane et al., 2001) and to quantify and counteract response bias (e.g., DeLeon et al., 2000).

In Lambert et al. (2024), RRE was a matching variable (i.e., a variable used to pair similar participants prior to randomization) that allowed those authors to control for fluctuations in reinforcer value through randomization. Thus, the isolated effects of reinforcer value on persistence during extinction could not be detected through that study's methods. Notwithstanding, alternative methods (i.e., *t* tests of logarithmically transformed Pearson correlation coefficients) allowed Lambert et al. (2026) to later establish that RRE was significantly correlated with equilibrium points produced by various unit prices during demand curve analysis (i.e., noncontingent reinforcement [NCR], FR 1, and *P*_*max*_) and was a better overall predictor of equilibrium than *P*_*max*_. In line with this finding, substance‐use research has demonstrated that measures of equilibrium when consumption was free of constraint (i.e., during NCR) and at optimal pricing (*P*_*max*_) could be predictive of treatment success for certain types of intervention programs (e.g., Mackillop et al., 2016; Murphy et al., 2015). Specifically, participants who consumed larger quantities of alcohol (Murphy et al., 2015) and nicotine (Mackillop et al., 2016) when it was freely available or who worked harder to defend consumption patterns in the face of rising costs were also more likely to relapse when competing incentives were arranged to disrupt consumption.

To the extent to which RRE serves as an index of response persistence, its quantification could allow practitioners to determine whether extinction is likely to be an effective treatment option prior to its implementation. Given the established relation between BP1 and equilibrium across a range of circumstances and the fact that PRAs could feasibly be embedded into current standard‐of‐practice procedures (i.e., functional analysis), this project sought to determine whether BP1 is significantly correlated with persistence in extinction. We also explored correlations with less effortful assessments (i.e., assessments of demand intensity obtained during NCR and FR 1) to determine whether either offered more explanatory power than BP1.

## METHOD

All hypotheses and methods for this study were formalized and preregistered in the Open Science Framework (Foster & Deardorff, 2017) prior to data analysis. Supporting Information A, B, and C include the preregistration template, planned analyses, and relevant R codes, respectively. Some of the data analyzed in this study (i.e., extinction data for 69 participants) were first published in Lambert et al. (2024). Although the analysis for this study was planned at project conceptualization (i.e., prior to Lambert et al., 2024), the specific analytic methods were not formalized and preregistered until after all data had been obtained. Notwithstanding, all obtained data had not been analyzed for the purposes articulated in this study prior to preregistration.

### Participant characteristics and sample size

Across a 2‐year period, we recruited and obtained consent for 111 adults with disabilities to participate in this study. Individuals were eligible if they (a) were older than 18, (b) had a disability diagnosis under the Individuals with Disabilities Education Act (2004), (c) could physically interact with study materials in a manner consistent with the study's requirements (e.g., exhibiting sufficient motor skills to squeeze a clothespin), and (d) consented to participate prior to study onset. If participants could not legally consent, we obtained consent from guardians, and assent from participants was obtained before each session. We did not anticipate that biological variables (e.g., sex) would affect the study outcomes and therefore did not set criteria for such variables.

Of the 111 participants enrolled, 90 completed the study elements relevant to this project (18.92% attrition). Reasons for attrition included scheduling conflicts or loss of interest (*n* = 16), health concerns unrelated to this study (*n* = 2), and fidelity errors during extinction challenges (*n* = 2). One participant moved to another home prior to study completion. In total, 90 participants met the eligibility criteria and completed all necessary phases for this study.

Supporting Information D includes participant demographic information. Of the participants who completed the study, 54.44% (49 of 90) identified (or were identified) as cisgender men, 43.33% (39 of 90) identified (or were identified) as cisgender women, 1.11% (1 of 90) identified as a transgender man, and 1.11% (1 of 90) identified as a transgender woman. Verbal reports indicated that 88.89% (80 of 90) of the participants were White, 6.67% (6 of 90) participants were Black or African American, 2.22% (2 of 90) of the participants were Asian (Indian), 1.11% (1 of 90) of the participants were American Indian or Alaska Native, and 1.11% (1 of 90) of the participants reported “other.”

The mean age of the participants was 40.3 years (range: 18–78), with a median of 35 years. The disabilities of 73 participants fell under a single category of the IDEA classification system, the disabilities of 14 participants spanned two IDEA categories, and the diagnoses of three participants spanned three IDEA categories. Using the IDEA classification system, 75 participants were reported to have an intellectual disability, 16 participants were reported to be autistic, 10 were reported to have other health impairments, two were reported to have orthopedic impairments, three were reported to have traumatic brain injuries, two were reported to have speech or language impairments, and two were reported to have visual impairments.

We chose to work with individuals with disabilities because this population is disproportionately affected by chronic challenging behavior (Bowring et al., 2019; Emerson et al., 2001; Simó‐Pinatella et al., 2019). Thus, we reasoned that exploring methods to circumvent the negative collateral effects of extinction could offer a benefit to this population. Understanding the potential for exploitation, especially given that our study was translational in nature, we chose to work with consenting adults so that we could compensate them for their time. For similar reasons, we took care to ensure participants enjoyed frequent and high‐quality social interactions with engaged research personnel in a reinforcing context. Supporting Information E includes the appointment mechanics. We also embedded a number of protections into our study procedures and recruitment efforts intended to ensure constant supervision and oversight from endogenous care providers unaffiliated with and uninvested in our research. All procedures were reviewed and approved by multiple institutional review boards as well as key stakeholders from all partnering organizations.

### Recruitment, consent, and settings

Recruitment materials were disseminated through seven organizations serving adults with disabilities across two states. Of the four organizations with interested clients, three specialized in residential and day‐service programming and one specialized in inclusive postsecondary education in a university setting. All recruitment efforts were mediated by third‐party representatives unaffiliated with the study who had a professionally established investment in the well‐being of prospective participants (e.g., program directors). That is, researchers never made initial contact with prospective participants and only contacted those whose interests had been vetted and approved by said mediators.

When interest was expressed, researchers scheduled a 30‐ to 45‐min meeting with each prospective participant (and/or their conservator). During this meeting, researchers explained that they were interested in learning how prospective participants “valued” reinforcers and that this could be learned by identifying the circumstances under which participants chose to stay at a table and work for a reinforcer selected by them. We clarified that participants would never be forced to participate and that our findings would not be valid if participants ever felt compelled to work for any reason other than to obtain the selected reinforcer. After answering all residual study‐related questions (e.g., study goals, study methods, compensation for participation), we initiated the informed consent process.

The consenting process entailed providing a written copy of the consent document and vocally describing each section of the form. When speaking with a participant's conservator, we asked whether the participants themselves could also consent. If they could, we completed the same process with them. If they could not, we asked the conservator to describe reliable indicators of both assent and dissent (e.g., approach responses, precursors to challenging behavior) and used those indicators to guide when and how all study activities were implemented. After obtaining written signatures confirming consent, an appointment schedule was developed according to convergent participant–researcher availability. Because study activities were not directly beneficial to participants, we only scheduled appointments at times not committed to work, instructional programming, or preferred leisure activities.

Sessions were conducted at the organizations from which participants were recruited (day program, workshop, or university settings for 71.11%, [64 of 90] of participants; home settings for 28.89% [26 of 90] of participants). These organizations maintained their business‐as‐usual staffing ratios and protocols, monitored study appointments (when needed), and maintained responsibility for all behavioral programming (when relevant). Sessions were conducted in a convenient location in these settings, with the participant seated at a table in an area visually accessible to endogenous stakeholders (e.g., staff, parents).

### Target response definitions, data recording, and dependent variables

Target responses were intentionally arbitrary to decrease the probability that response–reinforcer relations between them and programmed consequences had been established prior to study onset. They were also intentionally simple so that individuals with severe disabilities could have the option to participate. For 88 participants, target responding entailed die rolling (i.e., picking up a die and putting [or dropping] it back down). For the remaining two participants, target responding entailed manipulating a clothespin (i.e., picking up a clothespin, squeezing it, and putting it back down). Trained observers collected frequency data on these target responses. They also recorded the FR response requirements for each reinforcer, reinforcer delivery events, and overall session duration. Reinforcer delivery entailed either placing an edible item on a plate situated in front of participants (when relevant) or turning on a preferred sound for 30 s (when relevant). During PRA (described in procedures section), we recorded BP1 by documenting the schedule value of the last obtained reinforcer prior to response cessation (e.g., FR 1, FR 4, or FR 7). During the progressive FR analysis (PFRA; described in procedures section), we recorded the total number of reinforcers consumed during the first two conditions of the assessment (i.e., NCR, FR 1).

During baseline and extinction sessions of the extinction challenge, we calculated the mean baseline response rate (i.e., the mean rate of responding measured across all baseline sessions), the peak response rate during extinction (i.e., the response rate of a single extinction session, selected because it reflected the highest rate of responding observed during any extinction session), the peak response rate during extinction depicted as a proportion of baseline (i.e., the peak response rate divided by mean baseline response rate), total responses emitted during extinction, and the latency to extinction (i.e., elapsed time from extinction‐session onset to extinction‐session offset).

To probe the potential influence of the discriminability of contingency disruption on performance during extinction (e.g., Shahan, 2010, 2013, 2017), we also tracked the number of anticipated reinforcers “missed” prior to achieving an extinction effect. We did this by dividing the total number of responses emitted in extinction (e.g., 20) by the baseline schedule value (e.g., FR 4). That is, we quantified the number of times that participants should have expected to obtain a reinforcer but did not during extinction (e.g., 5).

### Interobserver agreement

We calculated interobserver agreement (IOA) for each dependent variable by comparing frequency counts scored by primary and secondary observers. This involved dividing the smaller count by the larger count and multiplying by 100 (e.g., Ledford & Gast, 2024). We then calculated a session agreement by aggregating IOA scores for each dependent variable. Across participants, the mean IOA for PRAs was 99.35% (range: 93%–100%) and was calculated for 33.21% (180 of 542) of PRA sessions. The mean IOA for PFRAs was 98.96% (range: 84.56%–100%) and was calculated for 42.12% (171 of 406) of PFRA sessions. The mean IOA for the extinction challenge was 99.14% (range: 81.33%–100%) and was calculated for 30.85% (311 of 1,008) of extinction‐challenge sessions.

### Procedural fidelity

Implementer fidelity evaluations entailed responses to yes or no checklists that described critical elements of session implementation. Supporting Information F–L include the procedural fidelity checklists. When a researcher implemented a session element as described, observers scored a “yes.” When they did not, observers scored a “no.” Session fidelity was then calculated by dividing all yes responses by the sum of yes + no responses and multiplying by 100. Across participants, mean fidelity for PRAs was 99.49% (range: 93.75%–100%) and was calculated for 94.46% (512 of 542) of PRA sessions. Mean fidelity for PFRAs was 99.76% (range: 95.23%–100%) and was calculated for 91.63% (372 of 406) of PFRA sessions. Mean fidelity for the extinction challenge was 99.75% (range: 90%–100%) and was calculated for 65.97% (665 of 1,008) of extinction‐challenge sessions.

### Experimental design

To generate a sample size large enough to explore correlations between relative reinforcer efficacy and persistence in extinction, we conducted a quasiexperimental prospective controlled consecutive case series (Hagopian, 2020). All participants completed assessments that could be used to establish a hierarchy of reinforcer efficacy (i.e., PRA, PFRA), followed by an extinction challenge. This sequence was selected because PRA outcomes served as the prerandomization matching variable in Lambert et al. (2024). The sequence was also selected because the PRA described by Reed et al. (2009)—our model for PRA design—called for three distinct exposures to their test condition. Thus, it was easy and convenient to intersperse control sessions into the PRA to efficiently establish experimental control of programmed consequences over target responding (via rapid iterative alternation; Bailey et al., 2024; Ledford et al., 2024).

To ensure sensitivity of the PRA assessment, we kept step sizes small, and unit price progression occurred in ascending order (Kaplan et al., 2018). Because PRAs can sometimes be experienced as aversive by participants (Leon et al., 2021; Poling, 2010), we attempted to attenuate any aversive qualities of our PRA procedures by clearly and frequently emphasizing their optional nature and by ensuring alternative reinforcers (e.g., access to other items, researcher attention) were always available for low‐effort alternative response options (Leon et al., 2021). We also continuously monitored assent and only implemented study procedures when it had clearly been secured (see procedures, below).

During the extinction challenge and as described in Lambert et al. (2024), we evaluated between‐groups differences for a subset of participant performances during a single exposure to extinction using a 2 × 2 factorial, crossed, and randomized matched blocks design, employing batched randomization logic. Specifically, we recruited participants in batches of 10 and matched two tetrads according to similarity in BP1. That is, we sorted participants into two groups of four pairings, which produced mean BP1 values with the smallest range of variation (relative to alternative participant combinations within the relevant group of 10). We then used the randomization functions of Microsoft Excel to assign members of each tetrad to one of four experimental groups—inelastic EO, inelastic AO, elastic EO, elastic AO.

Detailed descriptions of each condition can be found in the procedures section. Broadly, *inelastic* and *elastic* describe preextinction baseline conditions that served functions analogous to “rich” and “lean” baseline conditions in the behavioral momentum literature (e.g., Nevin & Grace, 2000). Likewise, EO and AO describe experimentally engineered fluctuations in motivating operations imposed immediately prior to extinction exposure. Thus, a participant randomized to the inelastic AO condition was exposed to an individualized rich baseline condition, followed by an extinction challenge that was immediately preceded by reinforcer consumption that approximated an individually determined equilibrium point. For contrast, a participant randomized to the elastic EO condition was exposed to an individualized lean baseline condition, followed by an extinction challenge, prior to which reinforcer consumption had been restricted.

The two participants not assigned to a tetrad were placed on hold after completing PRA and PFRA assessments until they could be matched with members of subsequently established tetrads (i.e., they did not advance to the extinction challenge until they had been randomly assigned to an experimental group). After enrollment for Lambert et al. (2024) was completed, an additional 22 participants were enrolled and assigned (without randomization) to a fifth condition (i.e., *P*_*max*_ EO). This final condition was added to determine whether transitions to extinction from optimally priced baseline schedules (i.e., *P*_*max*_) yielded different outcomes from transitions from suboptimally priced baseline schedules (i.e., inelastic, elastic). Due to resource constraints, we did not include a sixth, *P*_*max*_ AO, condition.

### Compensation

In addition to programmed reinforcers, all participants were compensated with gift cards at a rate commensurate with minimum wage in the states in which this study was conducted (i.e., $7.25/hr). To accomplish this, each participant was given a 10‐hole “punch card” (stored by researchers between appointments). Supporting Information M includes the punch card, and Supporting Information N includes the participant compensation tracking sheet. At the end of every appointment, researchers punched a hole in (or initialed) the card for every 20 min that the participant spent in the appointment. When all 10 holes had been initialed or punched, researchers immediately delivered a $25 gift card and initiated a new punch card during the next appointment.

Researchers always rounded up. For example, a participant who terminated an appointment after a 60‐s session would have earned one punch. A participant who terminated an appointment after 20 min and 60 s would have earned two punches. When participants could not manage their own money, gift cards were given to their conservators with the promise that the money would be spent to satisfy participants' preferences or needs.

### Procedures

For each participant, we scheduled one to five appointments per day across 1 to 5 days per week. To the extent possible, we held appointments at the same times each day and week to enhance consistency across MOs and other setting events. Similarly, we attempted to offer reinforcer options that were not typically available to participants (e.g., preferred snacks or activities not found in their homes). However, we did not ask caretakers to withhold preferred items or activities outside of formal study sessions. Thus, although we took steps to establish a closed economy, we could not guarantee it.

All participants completed the same progression: (a) interview (i.e., informed consent, appointment scheduling, identification of programmed reinforcers, and target responses), (b) target‐response training, (c) assessment of demand intensity (NCR), (d) PRA, (e) PFRA, and (f) extinction challenge. Researchers dedicated the first 5 min of every appointment toward rapport building (see “appointment mechanics”). To minimize unintended demand characteristics, all assessment‐related activities were framed as a choice. Specifically, researchers initiated each appointment with a 5‐min casual conversation. Prior to each session, researchers displayed the apparatus for the target response (e.g., dice) and a low‐preference alternative activity (e.g., a magazine or a toy) and paraphrased a script unique to each study condition (described below). The specific language for each script was shaped by problem‐solving efforts during pilot‐study initiatives as an attempt to preempt the generation of inaccurate self‐rules about prevailing contingencies or researcher expectations. All appointments terminated the moment a participant indicated they wanted it to end.

### Response training

All target responses entailed a multistep process (e.g., pick up a clothespin, squeeze it, put it down). The final step of each response (e.g., putting down a clothespin) automatically set up another opportunity to initiate it (e.g., picking up the clothespin). The target responses (die rolling or clothespin squeezing) were easy to execute and did not occur in the absence of our programmed consequences.

The target response topography was matched to each participant's skill set. During training, researchers modeled the correct response and instructed participants to do the same. If they could not, researchers used a least‐to‐most prompting procedure to assist initial responding and then delivered praise after each completion (Collins, 2022). If participants still could not independently emit the response, they were trained in the other response topography.

### Assessment of demand intensity

Demand intensity (i.e., reinforcer consumption during NCR) was assessed once. Facing an array of potential reinforcers, participants were asked to pick one that they would want to consume for the remainder of the study. The available options were in accord with participants' stated preferences and dietary restrictions. When the participant made their selection, researchers paraphrased the following statement:

We'll start working for (reinforcer) the next time I come. Right now, I'm just going to let you have as much of it as you want. Each time you take one and eat it [listen to it], I'll replace it with another. When you don't want to eat [listen] anymore, don't take anymore. Remember, you can always choose to play with this (low‐preference alternative) or tell me that you're done or that you'd rather talk to me than eat [listen].

For participants with complex communication needs, contingency reviews were simplified and experience based (i.e., responses were prompted and reinforced in conjunction with the delivery of key instructions).

For participants using edible reinforcers, researchers placed carefully measured and equal‐sized units of each food on a plate in front of the participant one at a time (after they made a choice and were given free access to their favorite). Each time participants picked up a morsel to consume, another was placed on the plate. Participants also had access to low‐preference alternative activities intended to compete with reinforcer consumption as the value of consumption depleted. Likewise, researchers did not speak to participants during sessions. If participants attempted to initiate a conversation while also consuming reinforcement, researchers indicated that they could not talk while participants were eating or listening to music but that they could talk if participants decided to terminate the session.

Sessions terminated when a participant refrained from consuming a reinforcer for 60 s (e.g., because they began to engage with the alternative activity), stated that they wished to terminate the session, touched a laminated “stop” card, or after 1 hr had elapsed (no assessment was ever terminated due to the passage of time). For participants with complex communication needs, we also accepted the act of walking away from the table as an indication of dissent or session termination.

Beyond the 1‐hr time limit, we did not constrain edible consumption during sessions. For participants with dietary restrictions, programmed consequences were auditory and reinforcement procedures were adapted from Lambert et al. (2019). That is, researchers began the session by playing the identified song on an iPhone. If the song finished before participants terminated a session, researchers allowed the next song of the relevant album to begin. Thus, participants could listen to up to an hour of music from the same album that contained the identified song. After session termination, we divided the session duration in seconds by 30 to determine the number of 30‐s units each participant had consumed during the assessment. To avoid confounds associated with satiation, no additional sessions were conducted during the same appointment in which this condition was conducted.

### Progressive‐ratio analysis

Participants were exposed to three PRA sessions, each interspersed with control sessions. Controls occurred prior to PRAs. To avoid satiation, only one test condition was conducted per appointment. Control and test conditions of the PRA were set up similarly to the assessment of demand intensity, with the following modifications.

#### Control

Researchers placed the target‐response apparatus (e.g., dice) in front of participants and delivered a paraphrase of the following contingency review:

We are going to start a session now. This just means I'm going to give you an opportunity to (task). When you do this (model task for participant), nothing is going to happen, so only do it if you like doing it. If it's boring, don't do it. During sessions, I cannot talk to you. That's just a rule I have to follow. It doesn't mean I don't want to talk or that I'm not happy. If you don't want to wait or would rather talk to me, you only need to stop (target response) for 1 min. You can also just say, “I'm done” or point to this stop card. While you wait, you can (alternative activity) if you like.

Researchers then began a session by saying, “3, 2, 1, start” and looking down to avoid eye contact. If participants emitted a target response during a control session, researchers interrupted additional responding and paraphrased the following statement:

Remember, right now I just need to know that you know that responding will not get you anything. The way that I'll know that is when you stop responding for 1 min. While you wait, you can play with (low‐preferred alternative). Or, if you're bored and don't want to wait anymore, you can say “all done” and we'll just end the session.

They then reset their timer and restarted the session. This continued until researchers obtained a 60‐s sample of the absence of target responding or participants asked to stop the session (we determined that both cases represented a disinclination to continue to emit the response in the absence of programmed consequences and provided evidence that responding that occurred during subsequently conducted test sessions was maintained by programmed consequences).

#### Test

Target responding during test sessions was reinforced according to a Basis 2 PRA 3 schedule. Specifically, after every other reinforcer delivery, price increased by three responses (e.g., FR 1, FR 4, and FR 7). At the beginning of each session, researchers paraphrased the following statement:

We are going to start a session, now. This just means I'm going to give you an opportunity to earn (reinforcer). At first it will be easy to earn (reinforcer), then it will take more time. During sessions, I cannot talk to you. That's just a rule I have to follow. It doesn't mean I don't want to talk or that I'm not happy. If you want (reinforcer) after the session starts, you can work on (target response). If you stop wanting (reinforcer), get too bored doing (task), or just want to talk to me, you only need to stop (target response) for 1 min. You can also just say, “I'm done” or point to this stop card. While you wait, you can (alternative activity) if you like.

Researchers then began a session by saying, “3, 2, 1, start” and looking down to avoid eye contact. If participants asked how many responses were needed to earn the next reinforcer, the researcher told them. For those who needed it, the researcher also counted out loud the number of responses made each time a new response was made. This was typically faded after delivery of the first three to five reinforcers of a given session.

Approximately once every 5 min, researchers reminded participants that they should not respond to make the researcher happy; that they should only continue responding if they wanted the programmed reinforcer; and that they should stop the moment they were tired of responding, didn't want the reinforcer, or simply wanted to quit. Sessions terminated when a participant refrained from emitting a target response for 60 s, stated that they wished to terminate the session, touched a laminated “stop” card, walked away from the table, or after 1 hr had elapsed (no session was ever terminated due to the passage of time).

### Progressive FR reinforcer analysis

This analysis was similar to the PRA with a few exceptions. First, the PFRA did not include a control condition. Next, schedule values were fixed (held constant) for the entire session. That is, response requirements increased by three across sessions rather than across reinforcer deliveries (this modification allowed us to establish equilibrium points for programmed reinforcers at each schedule value). To control for satiation, we conducted one PFRA session per appointment (sessions ended following 1 min without responding or after 1 hr). As was the case during the PRA, researchers then began each session by saying, “3, 2, 1, start” and looking down to avoid eye contact after paraphrasing the following contingency review.

In this version of the game, you can earn (reinforcer) by emitting the target response (assigned schedule value) number of times like this (researcher models response requirement). You can work for as long as you want to earn (reinforcer), I've bought a lot of them and am happy to keep giving them to you. But it's important that you only respond for as long as you want (reinforcer). The moment you're full, tired of responding, bored, or just want to talk, you just need to say you're done by touching this card. I'll pay you no matter what, so only respond for as long as you want (reinforcer). Can you show me that you know how to say you're done?

For participants who did not want to hear the entire contingency review, researchers simply asked them if they remembered what to do. If so, they asked participants to explain relevant contingencies to the researcher. If, during a session, participants asked how many responses were needed to earn the next reinforcer, the researcher told them. Likewise, researchers counted out loud the number of responses made by participants who needed this support for the first few reinforcer deliveries of a given session. Approximately once every 5 min, researchers also reminded participants that they could quit whenever they wanted to stop. Sessions terminated after 60 s elapsed without a target response, a participant stated that they wished to terminate the session, they touched a laminated “stop” card, they walked away from the table, or 1 hr had elapsed (no session was ever terminated due to the passage of time). The entire assessment ended after the aggregate response output for a current session fell below the output produced by the previous session (i.e., after *P*_*max*_ had been empirically confirmed).

### Extinction challenge (randomized controlled trial)

The purpose of the extinction challenge was to first establish a history with baseline contingencies of reinforcement. Baseline schedules were set according to PRA and PFRA results. Specifically, we compared the difference between each participant's PFRA‐identified *P*_*max*_ and the mean of their PRA‐identified BP1.

For participants assigned to the elastic condition, we added half of the calculated difference between *P*_*max*_ and BP1 to their *P*_*max*_ value and then rounded up to the nearest whole number. For example, if *P*_*max*_ was FR 4 and BP1 was FR 8, then half of the difference was two, which we added to *P*_*max*_ to establish an elastic baseline of FR 6. Similarly, for participants assigned to the inelastic condition, we subtracted half of the *P*_*max*_‐BP1 difference from *P*_*max*_ and rounded down to the nearest whole number (i.e., FR 2).

The default session duration for the baseline condition was 5 min. However, to ensure an opportunity to obtain no fewer than five reinforcers per session, we considered the latency to the first obtained reinforcer of the matched PFRA session (i.e., the PFRA session with a schedule value that most closely approximated the baseline schedule). For example, if the above‐mentioned participant was assigned to the elastic baseline condition (i.e., FR 6), we would consider their performance during the matched PFRA (FR 7) session to predict the number of obtained reinforcers possible during a typical 5‐min session. If, during the matched PFRA session, latency to the first obtained reinforcer was 56 s, we would conclude that it was physically possible for this participant to obtain at least five reinforcers at the default session duration of 5 min when the baseline reinforcement schedule was set at FR 6 and would thus not change schedule duration. However, if the latency to the first obtained reinforcer had been 90 s, we would have increased session duration to 7.5 min.

To set a maximum appointment duration (during which multiple consecutive baseline sessions could be conducted), we used the maximum duration of any previously recorded appointment as a proxy measure of AO (e.g., fatigue, satiation) and ensured appointment durations during the extinction challenge never exceeded that value by more than 5 min (i.e., the default duration of one baseline session). Similar considerations were made to ensure appointment‐reinforcer consumption never surpassed demand intensity (or the highest equilibrium point from the PFRA in cases in which NCR did not yield the highest consumption levels). For example, suppose the participant mentioned above consumed a maximum of 55 reinforcers during the FR 1 session of his PFRA. At FR 6 (his assigned baseline schedule), we would project that it would require 11 sessions to consume 55 reinforcers, estimating (roughly) 60 s to earn each reinforcer (justified above). Thus, this participant could have completed all 10 baseline sessions during a single appointment. In contrast, if participants could consume the maximum number of reinforcers during a single 5‐min session, then each of the 10 baseline sessions needed to be conducted across each of 10 distinct appointments. Notably, some participants completed more than one appointment per day. When this happened, researchers always ended the first appointment by crediting participants for their work and completing all relevant aspects of the compensation system (described above), allowing appointments to be interspersed with non‐study‐related activities and obtaining new assent for the subsequent appointment.

### Baseline

Baseline sessions were similar to PFRA sessions. During the first five sessions, researchers paraphrased the contingency review listed in the PFRA section (above) and reinforced target responding according to participant‐specific baseline schedules (i.e., inelastic, elastic). When allowable (i.e., when more than one baseline session could be conducted during an appointment and participants had not yet indicated they wanted to stop), researchers began new sessions immediately after previous sessions had terminated.

When participants terminated sessions early, we used the full 5‐min session duration as our denominator when calculating response rates. This was done to reflect the fact that participants were given the opportunity to respond across the full session duration, without artificially inflating rates of responding for participants who consistently terminated sessions early. That is, this decision allowed us to standardize our unit of analysis without requiring participants to remain in the research area after they had indicated they would like to leave.

### Extinction

During extinction, target responses ceased to produce programmed consequences. For AO participants, we introduced extinction after appointment‐reinforcer consumption fell one below the total number of reinforcers consumed during any single appointment or after a participant indicated they no longer wished to consume the reinforcer. Previous appointments were thus coordinated in such a way that extinction for the AO group was introduced toward the end of the relevant appointment (however, we did not cap appointment duration during extinction). If baseline sessions were completed before appointment‐reinforcer consumption levels were achieved, researchers delivered reinforcers one at a time and noncontingently to participants either until consumption fell one below demand intensity (or the highest equilibrium point from the PFRA in cases in which NCR did not yield the highest levels of consumption) or until participants indicated they wished to stop consuming reinforcers. In contrast, we introduced extinction to the EO group as the first session of the relevant appointment when consumption had been low.

In both cases (i.e., AO or EO) and prior to initiating the extinction session, researchers prompted participants to emit enough responses to earn a single reinforcer at the established baseline schedule value (e.g., FR 6) and then delivered a reinforcer. They then delivered the standard contingency review and reminded participants that they should stop when they got bored or did not want to consume more reinforcers.

Extinction continued until responding reduced to 10% (or less) of the mean of the final three baseline sessions across a duration equivalent to two consecutive sessions (i.e., 10 min) or until dissent. Importantly, to avoid disrupting the operation of extinction (or introducing other response‐suppressive elements attributable to procedures for transitioning between sessions), we conducted a single extinction session, which could last up to 1 hr. When the duration of extinction was greater than the duration of a single baseline session, we later segmented participant performance during extinction into intervals that matched baseline session duration (thus standardizing opportunity and allowing for direct comparisons between performances during baseline and extinction in graphical displays).

### Extinction challenge for *P* _*max*_ EO

This extinction challenge was initiated after the randomized controlled trial was completed and was conducted to explore whether or how extinction performances would be affected by baseline parameters set to *P*_*max*_ and EO. Baseline and extinction procedures in this condition were identical to those described above with the following exceptions: (a) baseline schedules were set according to PFRA‐observed *P*_*max*_ for all participants, (b) all extinction challenges followed procedures described for the EO group above (i.e., there were no assessments of persistence following AO exposures), or (c) participants were not randomly assigned to this group.

### Data analysis plan

Our regression models were designed to explore (a) whether and the extent to which RRE predicted response persistence during extinction and (b) which measure of RRE was most descriptive of persistence in extinction. We evaluated persistence in extinction using four measures—peak response rate, proportion of baseline, latency to extinction, and reinforcers missed (defined above).

We examined associations across three different measures of RRE (i.e., BP1, consumption at NCR, response output at FR 1). We used linear regression for each analysis, and the models controlled for independent variables known to be associated with response persistence—unit price relative to *P*_*max*_ (i.e., unit prices lower than *P*_*max*_ [inelastic unit prices]—unit prices higher than *P*_*max*_ [elastic unit prices]), and consumption relative to equilibrium (i.e., EO, AO). We also included interaction terms of each RRE measure with unit price and consumption relative to equilibrium. Thus, our initial model took the following form (note that the variable names in this equation differ in name only from the preregistration):

$$\text{Response}\_\text{persistence}=\beta_{0}+\beta_{1}\left(\mathrm{RRE}\right)+\beta_{2}\left(\mathrm{EO}\right)+\beta_{3}\left(\text{Elastic}\right)+\beta_{4}\left(P_{\text{max}}\right)+\beta_{23}\left(\mathrm{EO}\times \text{Elastic}\right)+\beta_{12}\;\left(\mathrm{RRE}\times \mathrm{EO}\right)+\beta_{13}\left(\mathrm{RRE}\times \text{elastic}\right)+\beta_{14}\;\left(\mathrm{RRE}\times P_{\text{max}}\right).$$

These were repeated once for each of the four response‐persistence outcomes: rate, proportion of baseline, latency, and reinforcers missed, and with each of the three measures of RRE: BP1, consumption at NCR, and consumption at FR 1, for a total of 12 statistical models. In these models, we were primarily interested in the effects of the RRE, including the size and statistical significance of the interactions (β12, β13, and β14). Note that the consumption and unit price conditions were interacted only once despite there being three conditions of unit price (i.e., inelastic, elastic, *P*_*max*_). This is because *P*_*max*_ AO was not tested. Therefore, this one interaction term (β23) holds all necessary conditions for these contrasts.

### Outliers, exclusions, and exploratory analyses

We first examined the data for outliers and defined an outlying case as one that was more than three *SD* from the mean. Due to the nature of this type of data, we were interested in analyzing the data “as is,” with outliers included. As expected, we identified multiple outliers in each of the measures of response persistence.

After identifying these outliers, we decided that a nonparametric statistical approach would be a better fit for the data. Therefore, in addition to these preregistered regression models, we conducted an exploratory analysis. We reanalyzed the data using median regression, a special case of quantile regression that is robust to nonnormality in residuals (Koenker & Hallock, 2001). Given that several predictors were expected to be moderately to highly correlated, we examined a correlation matrix to assess potential multicollinearity. High multicollinearity can inflate standard errors and potentially lead to unstable regression estimates.

#### Median regression

After conducting our preregistered analysis, we determined that Gaussian (normal) distributions were not well suited for analyzing all of our dependent variables—thus rendering our original plan less than optimal for its intended purpose (i.e., to assess the extent to which fluctuations in RRE moderated persistence in extinction and to identify the measure of RRE most likely to describe persistence in extinction). Consequently, and as described above, we determined after identifying outliers in the data that a median regression, which is robust to nonnormality, would be a better fit to the data. In planning the median regression, we also took into account the results of the previous regression models. The regression models above included statistical interactions between RRE and study conditions. With these interactions in the model, we were unable to interpret the overall main effect contribution of each RRE to response persistence. Given that our research questions were concerned with this overall contribution, we chose to drop those interactions from the estimating model for this series of median regressions.

## RESULTS

We conducted a correlation‐matrix analysis to identify bivariate correlations between the various dependent and independent variables and determine whether unconsidered relations were hidden by a high variance inflation factor. Table 1 demonstrates that no two variables were correlated strongly enough to raise concerns of multicollinearity (i.e., at or above .70).

**TABLE 1:** Bivariate‐correlations‐produced correlation matrix analysis.

Variables considered	BP 1	FR 1	NCR	Rate	Latency	Prop. of baseline	Reinforcers missed	Price high	Price low	Price *P*_*max*_	Deprived
BP1	1.00	0.65	0.21	0.30	0.23	−0.05	0.24	0.11	−0.02	−0.10	−0.05
FR 1	–	1.00	0.42	0.22	0.20	−0.13	0.15	−0.05	−0.01	0.07	−0.01
NCR	–	–	1.00	0.01	0.10	−0.08	0.09	−0.10	0.12	−0.02	−0.05
Rate	–	–	–	1.00	0.48	0.59	0.50	0.03	−0.20	0.20	0.31
Latency	–	–	–	–	1.00	0.26	0.37	0.05	−0.25	0.22	0.34
Prop. of baseline	–	–	–	–	–	1.00	0.67	−0.18	0.01	0.20	0.32
Reinforcers missed	–	–	–	–	–	–	1.00	−0.18	0.12	0.07	0.27
Price high	–	–	–	–	–	–	–	1.00	−0.62	−0.44	−0.14
Price low	–	–	–	–	–	–	–	–	1.00	−0.43	−0.25
Price *P*_*max*_	–	–	–	–	–	–	–	–	–	1.00	0.45
Deprived	–	–	–	–	–	–	–	–	–	–	1.00

*Note*: BP = breakpoint; FR = fixed ratio; NCR = noncontingent reinforcement; Prop = proportion. Dashes mark out redundant comparisons. Absolute values of .70 or greater typically raise concerns of multicollinearity. No two variables were correlated strongly enough in this data set to raise this concern.

Supporting Information O includes aggregate summaries of our raw data, and Supporting Information P–S include the results of our preregistered analyses. Table 2 displays the variance explained in the baseline regression models (in terms of *R* ^2^) and in the three different competing measures of RRE (BP1, FR 1, NCR) for each of the four conceptualizations of response persistence: rate, latency, proportion of baseline, and reinforcers missed.

**TABLE 2:** Variance explained in the baseline regression models (no RRE) and in the three different competing measures of RRE for each of the four conceptualizations of response persistence: rate, latency, proportion of baseline, and reinforcers missed.

Model	Rate	Latency	Proportion of baseline	Reinforcers missed
No RRE	13.1%	16.1%	12.0%	13.0%
RRE = BP 1	28.7%	24.7%	17.9%	25.1%
RRE = FR 1	20.9%	23.5%	15.6%	17.6%
RRE = NCR	16.3%	24.5%	16.6%	17.3%

*Note*: RRE = relative reinforcer efficacy; BP = breakpoint; FR = fixed ratio; NCR = noncontingent reinforcement. Variance explained is reported as an *R* ^2^ statistic.

For context, the baseline linear regression models (no RRE) in Table 2 accounted for the isolated and combined effects of two moderators known to have influenced persistence in this data set (i.e., fluctuations in unit price and preextinction reinforcer consumption; Lambert et al., 2024). Subsequent regressions in Table 2 demonstrate improvements in variance explained afforded by also considering fluctuations in each of the measures of RRE. Although each measure of RRE improved the model's explanatory power, BP1 consistently outperformed FR 1 and NCR and more than doubled the baseline model's accuracy in describing response persistence when depicted as a rate.

### Exploratory analyses: Median regression

Supporting Information T–W include the full results of our median‐regression analyses. Table 3 summarizes the RRE coefficients obtained from each of those regression models. Across models, BP1 obtained from the PRA was a statistically significant predictor of peak response rate during extinction (estimate = 0.54, *p* < .01), as was reinforcer consumption during FR 1 (estimate = 0.20, *p* < .01); however, reinforcer consumption during NCR was not associated (estimate = 0.00, *p* = 1.0). We found a similar pattern for latency to extinction such that two measures of RRE were statistically significantly associated with latency: BP1 (estimate = 7.74, *p* < .01) and NCR (estimate = 0.64, *p* = .02). However, FR 1 was not (estimate = 1.28, *p* = .20). None of the assessments of RRE were associated with response persistence when peak response rate was depicted as a proportion of baseline responding. However, response persistence, considered in terms of reinforcers missed, had the same statistical significance findings as rate and latency. BP1 was statistically significantly associated with the number of reinforcers missed (estimate = 0.46, *p* < .01), as was FR 1 (estimate = 0.11, *p* = .03) but not NCR (estimate = 0.04, *p* = .42).

**TABLE 3:** Beta coefficients, standard errors, and *p* values for each RRE variable.

Model	Rate		Latency		Proportion of baseline		Reinforcers missed	
	Estimate (*SE*)	*p*	Estimate (*SE*)	*p*	Estimate (*SE*)	*p*	Estimate (*SE*)	*p*
RRE = BP1	0.54 (0.12)	<.01*	7.74 (2.11)	< .01*	0.00 (0.01)	.98	0.46 (0.17)	<.01*
RRE = FR 1	0.20 (0.06)	<.01*	1.28 (0.99)	.20	0.00 (0.00)	.77	0.11 (0.05)	.03*
RRE = NCR	0.00 (0.03)	1.00	0.64 (0.27)	.02*	0.00 (0.00)	.70	0.04 (0.06)	.42

*Note*: RRE = relative reinforcer efficacy; BP = breakpoint; FR = fixed ratio; NCR = noncontingent reinforcement; *SE* = standard error. Beta coefficients describe the relation between variables while also controlling for the effects of other known moderators. Data in this table were pulled from median regression analyses (full model estimates available in Supporting Information T–W).

Figures 1, 2, 3, 4 display scatterplots showing the relation between each of the three measures of RRE with the four measures of persistence in extinction. For each, the line of best fit represents the bivariate correlation, providing a visual indication of the strength of the relation. Each plot also includes metrics from the full models in which unit price, consumption, and interaction terms were also included. We report the beta estimate and corresponding *p* value of RRE from the full regression models. Plots shaded in gray represent nonsignificant RRE estimates from the full models—that is, when a one‐unit change in the RRE was associated with a nonsignificant change in persistence in extinction, controlling for the influence of the model's other known moderators (i.e., unit price and consumption). In contrast, plots in black indicate significant estimates from the full models.

**FIGURE 1:**
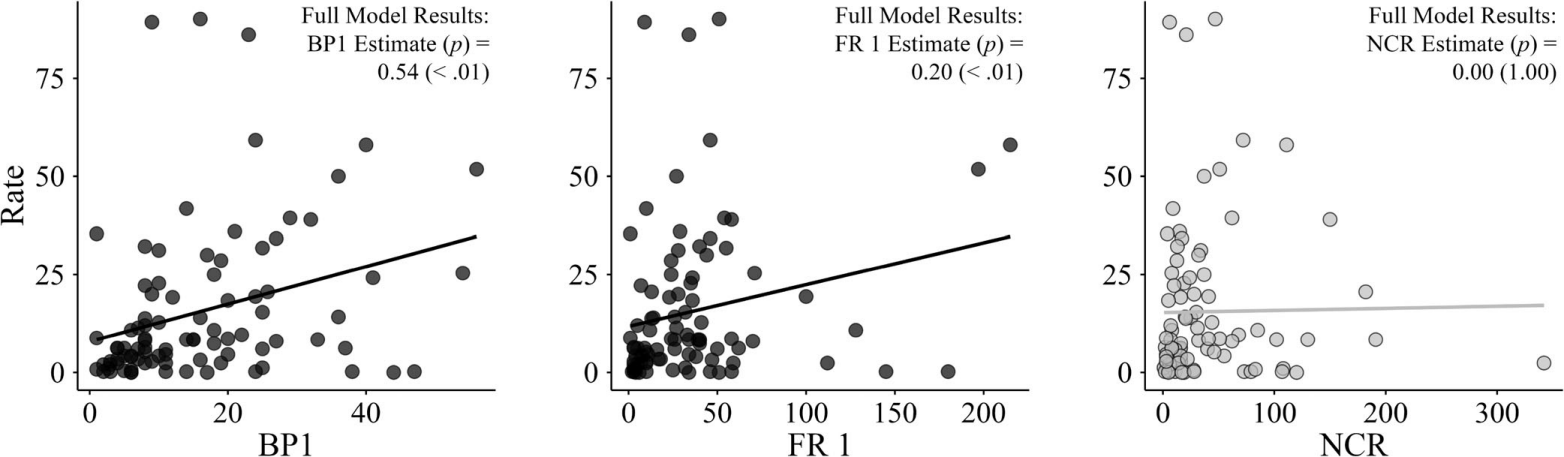
Scatterplots showing the relation between measures of RRE and response rate. Plots in gray indicate nonsignificant RRE estimates in full models predicting rate.

**FIGURE 2:**
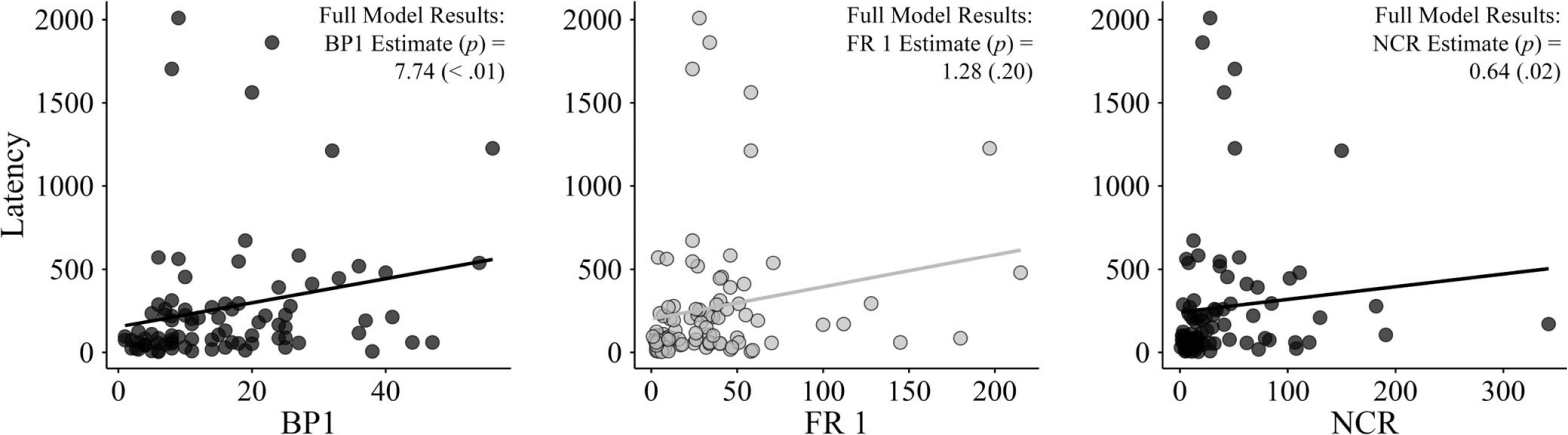
Scatterplot of RRE and latency. Plots in gray indicate nonsignificant RRE estimates in full models predicting latency.

**FIGURE 3:**
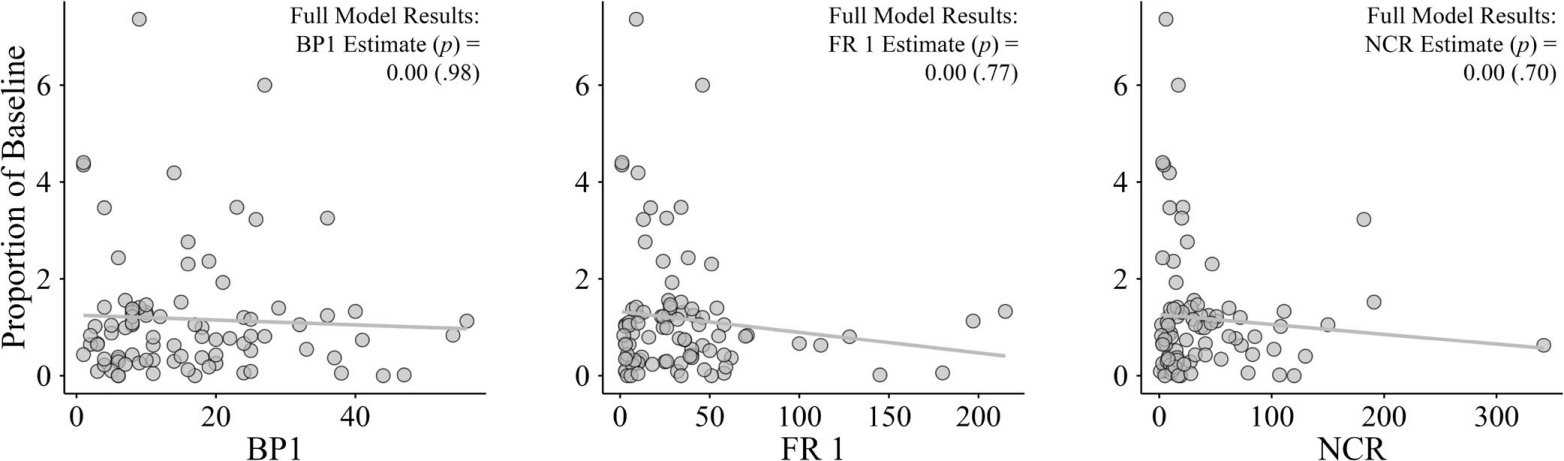
Scatterplot of RRE and proportion of baseline. Plots in gray indicate nonsignificant RRE estimates in full models predicting proportion of baseline.

**FIGURE 4:**
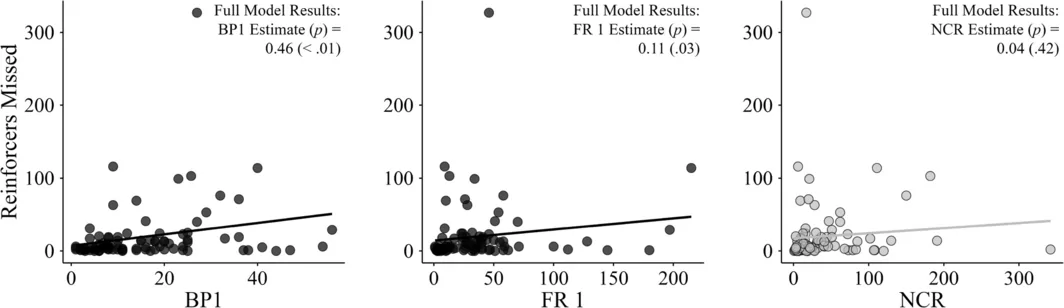
Scatterplot of RRE and reinforcers missed. Plots in gray indicate nonsignificant RRE estimates in full models predicting reinforcers missed.

Overall, there was substantial variability in the statistical significance of measures of RRE as predictors of measures of persistence in extinction. As indicated in Figure 1, BP1 and FR 1 were significantly associated with rate, whereas NCR was not. As indicated in Figure 2, BP1 and NCR were significantly associated with latency, whereas FR 1 was not. As indicated in Figure 3, no RRE was significantly associated with proportion of baseline. As indicated in Figure 4, BP1 and FR 1 were significantly associated with reinforcers missed, whereas NCR was not.

## DISCUSSION

There is some incongruity between concerns about side effects of extinction and the reliability of the occurrence of problematic outcomes (Katz & Lattal, 2020, 2021; Lattal et al., 2020; Nist & Shahan, 2021). Concerns regarding the anticipated negative collateral effects of extinction have led some researchers to advocate for the development of methods for achieving therapeutic reductions in challenging behavior that do not include extinction (MacNaul & Neely, 2018; Rajaraman et al., 2022; Trump et al., 2020). Although reinforcement‐based procedures that do not include extinction can sometimes be effective (e.g., Briggs et al., 2019; Kunnavatana et al., 2018; Staubitz et al., 2022), the efficacy of reinforcement‐based interventions often depends on contact with extinction (e.g., Hagopian et al., 1998; Lambert, Torelli, et al., 2021; Saini et al., 2017; Wacker et al., 1990).

Consequently, principled avoidance of extinction risks disallowing effective treatment to individuals who may require it and fails to take into consideration important contextual factors (e.g., the costs of ineffective intervention). One variable that could inform the viability of extinction is the extent to which challenging behavior persists after extinction is encountered. That is, practitioners may be more likely to abandon the intervention when challenging behavior is persistent. Thus, assessments that reliably predict persistence could hold prescriptive value for treatment‐design efforts.

As reinforcer value can moderate persistence in extinction (Lambert et al., 2024) and given data suggesting that measures of persistence may be related to reinforcer value (e.g., Livingston et al., 2025; Rickard et al., 2009; Sheehan et al., 2026; Taylor et al., 2025), it stands to reason that standard‐of‐practice procedures intended to identify and capitalize on reinforcer value (e.g., functional analysis) might also be capable of predicting persistence. However, during functional analysis, programmed reinforcers are delivered on FR 1 schedules (Lambert & Boyle, 2024; e.g., Rispoli et al., 2025; Sullivan et al., 2026). Because response rates maintained by reinforcers on FR 1 schedules are often poor predictors of either reinforcer preference or value when schedule requirements begin to approximate those representative of typical contexts (DeLeon et al., 1997; Francisco et al., 2008; Penrod et al., 2008), functional analysis methodology would likely need to be adapted before it could either establish a valid hierarchy of reinforcer value or predict persistence in extinction.

Using the logic and empirical precedent outlined in this article as justification, we hypothesized that embedding assessments of RRE into functional analysis (e.g., progressive‐ratio functional analysis) could be a viable and valid adaptation to the methodology that would allow researchers and practitioners to efficiently enhance treatment precision during the assessment process (e.g., Falligant & Hagopian, 2020; Hagopian et al., 2018; Weber et al., 2024). As a first step in testing this hypothesis, we explored correlations between RRE and the magnitude and persistence of responding using a low‐stakes translational model of the persistence of challenging behavior during extinction (for a comparable approach, see commentaries offered by Bloom & Lambert, 2015; Fuhrman et al., 2022; Lambert, 2026, and the associated iterative empirical contributions of Banerjee et al., 2022; Lambert et al., 2015, 2017, 2020; Lambert, Lamers, et al., 2021). Because PRAs require technical expertise and precision, we also explored correlations between response persistence and less effortful assessments (i.e., reinforcement consumption patterns during NCR and FR 1).

Of the measures tested, PRA‐derived BP1 was the most descriptive of persistence in extinction across the largest number of measures of that construct (i.e., rate, latency, and reinforcers missed but not proportion of baseline). Furthermore, BP1 sometimes doubled the variance accounted for by regression models that had already accounted for the individual and combined effects of fluctuations in baseline unit prices and fluctuations in MOs attributable to deprivation (i.e., the most commonly studied moderators of persistence in basic and translational behavior science).

However, despite statistically significant correlations, the combined effect of quantifying and controlling for unit price, fluctuations in MO, and all reported measures of RRE resulted in a regression model that never accounted for more than 30% of the variance in the obtained data set. Although this level of control is unprecedented in human operant literature exploring moderators of persistence in extinction, it is a far cry from the level needed to be clinically useful. Consequently, our findings do not justify embedding assessments of RRE (e.g., PRA) into standard‐of‐care assessments (e.g., functional analysis).

This conclusion is corroborated, to some extent, by basic research that has demonstrated an inverse relation between PRA outcomes and persistence (e.g., Forouzan et al., 2021; Hitchcock et al., 2019; Skjoldager, 1993) and by research that has challenged the construct validity of other common interpretations of PRA data (e.g., Lambert et al., 2026; Leon et al., 2021). To further complicate matters, there is some evidence to suggest that exposure to PRA methods can both increase persistence (Tsibulsky, 2021) and mitigate reinstatement (Kranak et al., 2022)—a form of relapse functionally linked to persistence (Podlesnik & Shahan, 2009, 2010). The threat of testing effects paired with statistically significant but underwhelming correlations reported here and in other research (outlined above), appears to dissuade further exploration.

A few of this study's limitations should be noted. First, we worked with members of a protected population to answer a research question not directly tethered to their clinical needs. Notwithstanding, our decision to work with this population was intentional and approved by multiple institutional review boards. Understanding the potential for exploitation, we compensated participants for their time using a scale commensurate with gainful employment and took care to ensure participants enjoyed frequent and high‐quality social interactions with engaged research personnel in a reinforcing context (see “appointment mechanics”). Anecdotally, some participants expressed pride in their contribution to science and most were overtly happy to “earn a paycheck.” Conservators of participants with more severe disabilities expressed gratitude that we had designed our study in a way that allowed individuals in their care to be included. All participants assented to every study session and appeared to enjoy the individualized reinforcement experiences that we contrived for them. Consequently, the ethical risks associated with including vulnerable populations in research seemed to us to be mitigated in this study.

Second, although participants were randomized to inelastic EO, inelastic AO, elastic EO, and elastic AO conditions, participants assigned to the *P*_*max*_ EO condition were not randomized. This diminishes the extent to which we can confidently suggest there was parity across experimental groups. Finally, this study targeted the persistence of arbitrary responses that had relatively short histories with programmed consequences.

Despite limitations, this project retains value because it demonstrates the practical limitations of a contemporary understanding of moderators of persistence and discourages the integration of highlighted technologies until additional conceptual and empirical work can be done to enhance model precision. Said more plainly, we do not recommend that researchers integrate PRA and FA methodology for the purpose of predicting the persistence of actual challenging behavior.

## AUTHOR CONTRIBUTIONS

The first author contributed to the conceptualization (equal), funding acquisition (lead), investigation (lead), methodology (equal), project administration (lead), resources (lead), supervision (lead), visualization (equal), writing the original draft (lead), and review and editing (equal). The second author contributed to the conceptualization (support), methodology (support), project administration (support), resources (support), visualization (equal), and review and editing (equal). The third author contributed to the conceptualization (support), methodology (equal), project administration (support), resources (support), visualization (support), and review and editing (support).

## CONFLICT OF INTEREST STATEMENT

There is no conflict of interest to declare.

## ETHICS APPROVAL

This study was preregistered, adhered to ethical guidelines, and was approved by Vanderbilt's institutional review board as well as the Utah State Department of Human Services institutional review board. Informed consent was obtained from all participants and data have been anonymized to protect participant privacy and confidentiality.

## Supporting information


**Supporting Information A** Preregistration Template from AsPredicted.org
**Supporting Information B** Planned Analyses: Preregistered Analytic Models Used for RRE Paper: Relative Reinforcer Efficacy Predicts Persistence in Extinction
**Supporting Information D** Individual Participant Demographic Information and Appointment Details
**Supporting Information E** Appointment Mechanics
**Supporting Information F** Procedural Fidelity Checklist for Free Access to Target Reinforcer
**Supporting Information G** Procedural Fidelity Checklist for Target Response Training
**Supporting Information H** Procedural Fidelity Checklist for Progressive Ratio Analysis (PRA) Control
**Supporting Information I** Procedural Fidelity Checklist for Progressive Ratio Analysis (PRA) Test
**Supporting Information J** Procedural Fidelity Checklist for Progressive Fixed‐Ratio Analysis (PFRA) Test
**Supporting Information K** Procedural Fidelity Checklist for Extinction Challenge (Baseline)
**Supporting Information L** Procedural Fidelity Checklist for Extinction Challenge (Extinction)
**Supporting Information M** Punch Card
**Supporting Information N** Participant Compensation Tracking Sheet
**Supporting Information P** Results for the Models Predicting Response Rate During Extinction (preregistered analysis)
**Supporting Information Q** Results for Models Predicting Latency to Extinction (preregistered analysis)
**Supporting Information R** Model Results Predicting the Response as a Proportion of Baseline, Such That Peak Response Rate is Divided by the Mean Response Rate for Each Responder (preregistered analysis)
**Supporting Information S** Model Results Predicting the Anticipated Number of Reinforcers Missed Prior to Achieving an Extinction Effect (preregistered analysis)
**Supporting Information T** Median Regression Results Predicting Rate from Conditions of the Trial and Competing RRE
**Supporting Information U** Median Regression Results Predicting Latency from Conditions of the Trial and Competing RRE
**Supporting Information V** Median Regression Results Predicting Proportion of Baseline from Conditions of the Trial and Competing RREs
**Supporting Information W** Median Regression Results Predicting Reinforcers Missed from Conditions of the Trial and Competing RRE

Supporting information C

Supporting information O

## Data Availability

The data that support the findings of this study are available from the corresponding author on reasonable request. Supporting Information [Link], [Link] offer our preregistration template, analyses, and relevant R codes. Supporting Information D offers individual participant demographic information, Supporting Information E offers information about our appointment mechanics, Supporting Information F–L include each procedural fidelity checklist, Supporting Information M and N include materials needed to track and compensate our participants, and Supporting Information O contains aggregate summaries of our raw data, Supporting Information P–S include the results of our preregistered analyses, and Supporting Information T–W include the results of our exploratory analyses.
