# Supplementary material for: Relative reinforcer efficacy and persistence in extinction are significantly correlated but not clinically actionable
Source: J Appl Behav Anal. 2026 Nov 8;60(1):e70097. doi: 10.1002/jaba.70097 (PMC13745635; doi:10.1002/jaba.70097)
Supplement: Supplementary file 1 [file JABA-60-0-s003.docx]

**Supporting Information A**

*Preregistration Template from AsPredicted.org*

**Data collection**

Has any data been collected for this study already? Note: 'Yes' is a discouraged answer for this preregistration form.

The data for this study have already been collected, in conjunction with a randomized control trial designed to explore the functionality of considering the economic concepts of elasticity and diminishing marginal utility when attempting to predict and control extinction bursts in adults with disabilities (i.e., Lambert et al., 2024). Although the analysis for *this* study was planned (in principle) at project conceptualization (i.e., prior to data collection), the specific analytic methods were not formalized until after all data had been obtained. This was the case due to a misalignment between the scope of competence of the first author (an intervention researcher with expertise in single case design), and the expertise needed to answer this question. Importantly, the data for this analysis have not previously been analyzed for the purposes articulated in this study.

**Hypothesis**

What's the main question being asked or hypothesis being tested in this study

This study aims to explore correlations between PRA-observed breakpoints and the magnitude and persistence of human responding when it contacts extinction. The identification of consistent and orderly relations between behavior and circumstance in analogue settings may serve as justification for the development of novel approaches to the assessment and treatment of challenging behavior (i.e., the primary driving force of the overarching project). That is, the ultimate objective of this study is to explore the extent to which there is empirical justification to develop deviations from established evidence-based practices (e.g., functional analysis) in the service of efficacy and social palatability.

Because PRA is an effortful analysis to conduct, we also aim to explore correlations between the magnitude and persistence of human responding when it contacts extinction and data obtained from less effortful assessments (i.e., reinforcement consumption patterns during NCR and FR 1).

Importantly, we do not have directional hypotheses. In this study, we ask under what circumstances do participants persist in a strategy when the strategy is ineffective? We are conceptualizing the outcome as responding during extinction, quantifying this as Rate, Latency, peak extinction-response rate displayed as a Proportion of Baseline response rate, and Number of Anticipated Reinforcers missed during extinction (prior to response cessation). For each analysis, we will control for baseline reinforcement parameters.

Controlling for condition (i.e., Price [low, high, or Pmax] and Deprivation [deprived or satiated]), we will examine the association between response during extinction (Rate, latency, proportion of baseline, and reinforcers missed) and Contingency Parameters of baseline assessments (PRA breakpoint, FR 1, and NCR).

**Conditions**

How many and which conditions will participants be assigned to?

Participants were randomly assigned to one of the four original experimental conditions through matched (according to PRA breakpoint) and blocked (every 10 participants, with the two outliers matched to participants from subsequent blocks) randomization procedures.

1. Low price (relative to Pmax) + Satiated
2. Low price (relative to Pmax) + Deprived
3. High price (relative to Pmax) + Satiated
4. High price (relative to Pmax) + Deprived

After all data for the RCT had been obtained, we collected additional data for a fifth condition in which participants weren’t randomized.

5. Pmax + Deprived

**Variables**

Describe the key variable(s) specifying how they will be measured.

For 88 participants, target responding entailed die rolling (i.e., picking up a die and putting [or dropping] it back down). For the remaining two participants, target responding entailed manipulating a clothespin (i.e., picking up a clothespin, squeezing it, and putting it back down). Trained observers collected continuous timed-event measures of the frequency of these target responses. They also recorded the FR response requirements for each reinforcer, reinforcer delivery events, and overall session duration. Reinforcer delivery entailed either placing an edible item on a plate situated in front of participants (when relevant) or turning on a preferred sound for 30 s (when relevant).

During progressive-ratio reinforcer analysis (PRA), we recorded the specific schedule value (e.g., FR 1, FR 4, FR 7), which was satisfied prior to each reinforcer delivery. During progressive fixed-ratio reinforcer analysis (PFRA), we recorded the specific schedule value (e.g., FR 1, FR 4, FR 7), which produced the highest response output (i.e., Omax).

Derivative measures appropriate to each analysis described below served as our dependent variables and included breakpoint-BP1 (i.e., the schedule of the last obtained reinforcer prior to response cessation [PRA]) and observed Pmax (the schedule value at which the maximum response output was obtained [PFRA]). During baseline and extinction sessions of the extinction challenge, we calculated mean baseline response rate, peak response rate during extinction (i.e., the rate of responding during the session that displayed the single highest rate of responding during extinction), peak response rate during extinction depicted as a proportion of baseline (i.e., the peak response rate divided by mean baseline response rate, multiplied by 100), total responses emitted during extinction, and latency to extinction (i.e., elapsed time from extinction-session onset to extinction-session offset).

To probe the potential impact of the discriminability of contingency disruption on performance during extinction, we also tracked reinforcers “missed” (e.g., five) prior to achieving an extinction effect by dividing the total number of responses emitted in extinction (e.g., 20) by the baseline schedule value (e.g., FR 4). That is, we quantified the number of times that participants should have expected to obtain a reinforcer but did not during extinction prior to desisting. This final variable was exploratory.

Other than conditional assignment, additional Independent Variables are the Contingency Parameters of pre-baseline assessments. Specifically, PRA-breakpoint (i.e., response patterns when reinforcer access increases as a function of consumption patterns), unconstrained reinforcer consumption (NCR), and reinforcer consumption at a low and constant unit price (FR 1).

**Analyses**

Specify exactly which analyses you will conduct to examine the main question/hypothesis.

Our primary research aim is to examine whether the relation between Contingency Parameters of baseline assessments (PRA breakpoint, FR 1, and NCR) and response during extinction (Rate, latency, proportion of baseline, and reinforcers missed) is moderated by conditions of the trial.

The models will take the following form:

p$Response = b_{o}+b_{1}\left( Bpoint \right) +b_{2}\left( Deprived \right)+b_{3}\left( Price high \right)+ b_{4}\left( Price pMax \right)+b_{23}\left( Deprived \right)\left( Price high \right)+b_{12}\left( Bpoint \right)\left( Deprived \right)+b_{13}\left( Bpoint \right)\left( Price high \right)+b_{14}\left( Bpoint \right)\left( Price pMax \right)$

These will be repeated once for each of the four outcomes of interest: Rate, Latency, Proportion of Baseline, and Reinforcers missed, and with each of the three conceptualizations of reinforcer value (PRA breakpoint, FR 1 consumption patterns, and NCR consumption patterns), for a total of 12 statistical models.

In these models, we are primarily interested in the effect sizes and statistical significance of the interactions (b12, b13, and b14).

Note that the Deprivation and Price conditions are interacted only once, despite there being three conditions of Price (low, high, or Pmax). This is because Pmax was not tested when participants were satiated. Therefore, this one interaction term (b23) holds all necessary conditions for these contrasts.

**Outliers and Exclusions**

Describe exactly how outliers will be defined and handled, and your precise rule(s) for excluding observations.

We will first examine the data for outliers and will define an outlying case as one that is more than three standard deviations from the mean. Due to the nature of this type of data, we are interested in analyzing the data “as-is”, with outliers included. We believe all data points are accurate and represent the participants’ true responses.

**Sample Size**

How many observations will be collected or what will determine the sample size? No need to justify the decision, but be precise about exactly how the number will be determined.

Across a two-year period, we recruited and consented 111 adults with disabilities to participate in this study. Individuals were eligible if they (a) were older than 18, (b) had a disability diagnosis under the Individuals with Disabilities Education Act (IDEA, 2004), (c) could exhibit the ability to physically interact with study materials in a manner consistent with the study’s requirements (e.g., exhibiting sufficient motor skills to squeeze a clothespin), and (d) consented to participate prior to student onset. If participants could not legally consent, we obtained consent from guardians and assent from participants was obtained before each session. We did not anticipate biological variables (e.g., sex) would impact study outcomes and therefore did not set criteria for such variables. Of the 111 participants enrolled, 90 completed the study elements relevant to this project (18.91% attrition). Reasons for attrition included scheduling conflicts and/or loss of interest (*n* = 16), health concerns unrelated to this study (*n* = 2), and fatal fidelity errors during the extinction challenge (n=2). One participant moved to another home prior to study completion.

In total, we have 90 participants who met eligibility criteria, after attrition, and who completed all necessary phases for our study.

**Other**

Anything else you would like to pre-register? (e.g., secondary analyses, variables collected for exploratory purposes, unusual analyses planned?)

All planned analyses are described in the previous sections.

**Name**

Give a title for this AsPredicted pre-registration. Suggestion: use the name of the project, followed by the study description.

Do Breakpoints Obtained from Progressive Ratio Analysis Predict Magnitude and Persistence During Extinction?

**Finally**

For record keeping purposes, please tell us the type of study you are pre-registering.

- Class project or assignment
- Experiment
- Survey
- Observational/archival study
- Other (describe below)

**Supporting Information B**

*Planned Analyses: Preregistered Analytic Models Used for RRE Paper: Relative Reinforcer Efficacy Predicts Persistence in Extinction*

---

title: "RRE analysis"

output: html_document

date: "2025-02-25"

---

```{r setup, include=FALSE}

knitr::opts_chunk$set(echo = TRUE)

```

Research Question:

Is the relation between contingency parameters of baseline (PRA breakpoints, FR 1, NCR) and response during extinction (measured as rate, latency, proportion of baseline, and reinforces missed) is moderated by conditions of the trial.

This reflects the preregistration.

Analysis Plan:

response = b0 + b1(bpoint) + b2(deprived) + b3(price high) + b4(price pmax) + b23(deprived)(price high) + b12(deprived)(bpoint) + b13(price high)(bpoint) + b14(price pmax)(bpoint)

These will be repeated once for each of the four outcomes of interest: Rate, Latency, Proportion of Baseline, and Reinforcers missed, and with each of the three conceptualizations of reinforcer value (PRA breakpoint, FR 1 consumption patterns, and NCR consumption patterns), for a total of 12 statistical models.

In these models, we are primarily interested in the effect sizes and statistical significance of the interactions (b12, b13, and b14).

Data and Libraries

```{r}

library(dplyr)

library(broom)

library(car)

library(readxl)

library(tidyr)

library(parameters)

library(ggplot2)

setwd("xxxx")

```

Data Cleaning/Wrangling

```{r}

head(df2) #check placement of col names

colSums(is.na(df2)) # check missing data per var

\# rename cols to match model equations

\## contingency(?)/breakpoints

df2 <- df2 %>% rename(PRA = `PRA Bpoint`)

df2 <- df2 %>% rename(FR 1 = `Reinforcers Consumed/Responses Emitted (PFRA FR 1)`)

df2 <- df2 %>% rename(NCR = `Reinforcers Consumed (PFRA NCR)`)

\## outcome

df2 <- df2 %>% rename(rate = `Ext Responding (Rate)`)

df2 <- df2 %>% rename(latency = `Latency to Extinction`)

df2 <- df2 %>% rename(prop_bl = `Ext Responding (Rate)-Proportion of BL`)

df2 <- df2 %>% rename(rein_missed = `Ext "Missed" Reinforcers`)

\# recode "Group Assignments" into 2 variables

df2 <- df2 %>%

mutate(

price_high_ref = case_when(

grepl("Cheap", `Group Assignment`) ~ 0,

grepl("Expensive", `Group Assignment`) ~ 1,

grepl("Pmax", `Group Assignment`) ~ 2

)

)

\# create 'deprived' depending on what is mentioned in 'Group Assignment'

df2 <- df2 %>%

mutate(

price_high = case_when(

grepl("Cheap", `Group Assignment`) ~ 0,

grepl("Expensive", `Group Assignment`) ~ 1,

grepl("Pmax", `Group Assignment`) ~ 2

), # create 'price_high' depending on what is mentioned in 'Group Assignment'

\# to be further changed

deprived = case_when(

grepl("EO", `Group Assignment`) ~ 1,

grepl("AO", `Group Assignment`) ~ 0

)

)

\# create 'deprived' depending on what is mentioned in 'Group Assignment'

\# recode price variable into 2 variables: cheap vs expensive vs NA and Pmax vs not Pmax

df2 <- df2 %>%

mutate(

price_pmax = ifelse(price_high == 2, 1, 0),

\# create 'pmax': 1 if price_high == 2, else 0

price_high = ifelse(price_high == 2, 0, price_high)

\# replace 2(=pmax) with NA in 'price_high'

)

```

New vars:

price_high:

cheap = 0

expensive = 1

pmax condition = 0

#** Note that we call these "price" , cheap, and expensive. In the paper, we refer to these as Elastic and Inelastic **

pmax:

yes pmax = 1

not pmax = 0

deprived:

EO = 1

AO = 0

#** Note that we call this variable "deprived" but in the equations in the paper we call this "EO" **

Check group means:

```{r}

mean(df2$rate)

sd(df2$rate)

\# AO vs EO

df2 %>%

group_by(deprived) %>%

summarise(mean(rate, na.rm = TRUE))

\# Cheap vs Expensive vs Pmax

df2 %>%

group_by(price_high) %>%

summarise(mean(rate, na.rm = TRUE))

\## Matrix

\# n per bin

table(df2$price_high, df2$deprived)

\# Compute mean rate for each combination (pivot is to make table easier to read)

df2 %>%

group_by(price_high_ref, deprived) %>%

summarise(mean_rate = mean(rate, na.rm = TRUE), .groups = "drop") %>% # Name the column

pivot_wider(names_from = deprived, values_from = mean_rate, names_prefix = "deprived_")

```

Baseline Models - No RRE included to establish percent variance explained from each model

```{r}

model0b <- lm(rate ~ deprived + price_high + price_pmax + price_high:deprived, data = df2)

summary(model0b)

model0c <- lm(latency ~ deprived + price_high + price_pmax + price_high:deprived, data = df2)

summary(model0c)

standardize_parameters(model0c)

model0d <- lm(prop_bl ~ deprived + price_high + price_pmax + price_high:deprived, data = df2)

summary(model0d)

standardize_parameters(model0d)

model0e <- lm(rein_missed ~ deprived + price_high + price_pmax + price_high:deprived, data = df2)

summary(model0e)

standardize_parameters(model0e)

```

#%%%%%%%%%%%%%%%%%%%%%%%%%%%%%%%%%%%%%%%%%%%%%%%%%%%%%%%%%%%%%%%%%%%%%%%%%%%%%%%

#%%%%%%%%%%%%%%%%%%%%%%%%%%%%%%%%%%%%%%%%%%%%%%%%%%%%%%%%%%%%%%%%%%%%%%%%%%%%%%%

Table 1	OUTCOME = Rate

#%%%%%%%%%%%%%%%%%%%%%%%%%%%%%%%%%%%%%%%%%%%%%%%%%%%%%%%%%%%%%%%%%%%%%%%%%%%%%%%

#%%%%%%%%%%%%%%%%%%%%%%%%%%%%%%%%%%%%%%%%%%%%%%%%%%%%%%%%%%%%%%%%%%%%%%%%%%%%%%%

#rate - Table 1

```{r}

model_interactions_c <- lm(rate ~ PRA + deprived + price_high + price_pmax + price_high:deprived + FR 1:deprived + FR 1:price_high + FR 1:price_pmax, data = df2)

summary(model_interactions_c)

model_interactions_d <- lm(rate ~ FR 1 + deprived + price_high + price_pmax + price_high:deprived + FR 1:deprived + FR 1:price_high + FR 1:price_pmax, data = df2)

summary(model_interactions_d)

model_interactions_e <- lm(rate ~ NCR + deprived + price_high + price_pmax + price_high:deprived + FR 1:deprived + FR 1:price_high + FR 1:price_pmax, data = df2)

summary(model_interactions_e)

```

#%%%%%%%%%%%%%%%%%%%%%%%%%%%%%%%%%%%%%%%%%%%%%%%%%%%%%%%%%%%%%%%%%%%%%%%%%%%%%%%

#%%%%%%%%%%%%%%%%%%%%%%%%%%%%%%%%%%%%%%%%%%%%%%%%%%%%%%%%%%%%%%%%%%%%%%%%%%%%%%%

Table 2 OUTCOME = Latency

#%%%%%%%%%%%%%%%%%%%%%%%%%%%%%%%%%%%%%%%%%%%%%%%%%%%%%%%%%%%%%%%%%%%%%%%%%%%%%%%

#%%%%%%%%%%%%%%%%%%%%%%%%%%%%%%%%%%%%%%%%%%%%%%%%%%%%%%%%%%%%%%%%%%%%%%%%%%%%%%%

#latency - Table 2

```{r}

Model2_interactions_a <- lm(latency ~ PRA + deprived + price_high + price_pmax + price_high:deprived + PRA:deprived + PRA:price_high + PRA:price_pmax, data = df2)

summary(Model2_interactions_a)

Model2_interactions_b <- lm(latency ~ FR 1 + deprived + price_high + price_pmax + price_high:deprived + FR 1:deprived + FR 1:price_high + FR 1:price_pmax, data = df2)

summary(Model2_interactions_b)

Model2_interactions_c <- lm(latency ~ NCR + deprived + price_high + price_pmax + price_high:deprived + NCR:deprived + NCR:price_high + NCR:price_pmax, data = df2)

summary(Model2_interactions_c)

```

#%%%%%%%%%%%%%%%%%%%%%%%%%%%%%%%%%%%%%%%%%%%%%%%%%%%%%%%%%%%%%%%%%%%%%%%%%%%%%%%

#%%%%%%%%%%%%%%%%%%%%%%%%%%%%%%%%%%%%%%%%%%%%%%%%%%%%%%%%%%%%%%%%%%%%%%%%%%%%%%%

Table 3 OUTCOME = Proportion of Baseline

#%%%%%%%%%%%%%%%%%%%%%%%%%%%%%%%%%%%%%%%%%%%%%%%%%%%%%%%%%%%%%%%%%%%%%%%%%%%%%%%

#%%%%%%%%%%%%%%%%%%%%%%%%%%%%%%%%%%%%%%%%%%%%%%%%%%%%%%%%%%%%%%%%%%%%%%%%%%%%%%%

#prop_bl

```{r}

Model3_interactions_a <- lm(prop_bl ~ PRA + deprived + price_high + price_pmax + price_high:deprived+ PRA:deprived + PRA:price_high + PRA:price_pmax, data = df2)

summary(Model3_interactions_a)

Model3_interactions_b <- lm(prop_bl ~ FR 1 + deprived + price_high + price_pmax + price_high:deprived+ FR 1:deprived + FR 1:price_high + FR 1:price_pmax, data = df2)

summary(Model3_interactions_b)

Model3_interactions_c <- lm(prop_bl ~ NCR + deprived + price_high + price_pmax + price_high:deprived+ NCR:deprived + NCR:price_high + NCR:price_pmax, data = df2)

summary(Model3_interactions_c)

```

#%%%%%%%%%%%%%%%%%%%%%%%%%%%%%%%%%%%%%%%%%%%%%%%%%%%%%%%%%%%%%%%%%%%%%%%%%%%%%%%

#%%%%%%%%%%%%%%%%%%%%%%%%%%%%%%%%%%%%%%%%%%%%%%%%%%%%%%%%%%%%%%%%%%%%%%%%%%%%%%%

Table 4 OUTCOME = Reinforcers Missed

#%%%%%%%%%%%%%%%%%%%%%%%%%%%%%%%%%%%%%%%%%%%%%%%%%%%%%%%%%%%%%%%%%%%%%%%%%%%%%%%

#%%%%%%%%%%%%%%%%%%%%%%%%%%%%%%%%%%%%%%%%%%%%%%%%%%%%%%%%%%%%%%%%%%%%%%%%%%%%%%%

#rein_missed

```{r}

Model4_interactions_a <- lm(rein_missed ~ PRA + deprived + price_high + price_pmax + price_high:deprived+ PRA:deprived + PRA:price_high + PRA:price_pmax, data = df2)

summary(Model4_interactions_a)

Model4_interactions_b <- lm(rein_missed ~ FR 1 + deprived + price_high + price_pmax + price_high:deprived+ FR 1:deprived + FR 1:price_high + FR 1:price_pmax, data = df2)

summary(Model4_interactions_b)

Model4_interactions_c <- lm(rein_missed ~ NCR + deprived + price_high + price_pmax + price_high:deprived+ NCR:deprived + NCR:price_high + NCR:price_pmax, data = df2)

summary(Model4_interactions_c)

```

###################################################################################\

Next, Exploratory analyses due to outliers, we check whether this is robust with nonparametric statistical approaches:

###################################################################################\

```{r}

library(quantreg)

\## Table 6 : RATE

qr_1a = rq(rate ~ PRA + deprived + price_high + price_pmax + price_high:deprived

, data = df2,

tau = 0.5)

qr_1b = rq(rate ~ FR 1 + deprived + price_high + price_pmax + price_high:deprived

, data = df2,

tau = 0.5)

qr_1c = rq(rate ~ NCR + deprived + price_high + price_pmax + price_high:deprived

, data = df2,

tau = 0.5)

summary(qr_1a,se = "nid")

summary(qr_1b,se = "nid")

summary(qr_1c,se = "nid")

\## Table 7: LATENCY

qr_2a = rq(latency ~ PRA + deprived + price_high + price_pmax + price_high:deprived

, data = df2,

tau = 0.5)

qr_2b = rq(latency ~ FR 1 + deprived + price_high + price_pmax + price_high:deprived , data = df2,

tau = 0.5)

qr_2c = rq(latency ~ NCR + deprived + price_high + price_pmax + price_high:deprived , data = df2,

tau = 0.5)

summary(qr_2a,se = "nid")

summary(qr_2b,se = "nid")

summary(qr_2c,se = "nid")

\## Table 8: PROPORTION OF BASELINE

qr_3a = rq(prop_bl ~ PRA + deprived + price_high + price_pmax + price_high:deprived, data = df2,

tau = 0.5)

qr_3b = rq(prop_bl ~ FR 1 + deprived + price_high + price_pmax + price_high:deprived, data = df2,

tau = 0.5)

qr_3c = rq(prop_bl ~ NCR + deprived + price_high + price_pmax + price_high:deprived, data = df2,

tau = 0.5)

summary(qr_3a,se = "nid")

summary(qr_3b,se = "nid")

summary(qr_3c,se = "nid")

\## Table 9: REINFORCERS MISSED

qr_4a = rq(rein_missed ~ PRA + deprived + price_high + price_pmax + price_high:deprived, data = df2,

tau = 0.5)

qr_4b = rq(rein_missed ~ FR 1 + deprived + price_high + price_pmax + price_high:deprived, data = df2,

tau = 0.5)

qr_4c = rq(rein_missed ~ NCR + deprived + price_high + price_pmax + price_high:deprived, data = df2,

tau = 0.5)

summary(qr_4a,se = "nid")

summary(qr_4b,se = "nid")

summary(qr_4c,se = "nid")

```

**Supporting Information D**

*Individual Participant Demographic Information and Appointment Details*

| **Participant** | **Age** | **Race** | **Ethnicity** | **Gender** | **Disability** | **S^R+^** | **Target R** | **1^0^ Appt Setting** |
| --- | --- | --- | --- | --- | --- | --- | --- | --- |
| P1 | 54 | White | Non-Hispanic | Male | ID; VI | Snack | Clothespin | Home |
| P2 | 50 | White | Non-Hispanic | Female | ID | Snack | Dice Roll | Home |
| P4 | 55 | White | Non-Hispanic | Female | ID | Snack | Dice Roll | Home |
| P5 | 57 | Black | Non-Hispanic | Female | ID | Snack | Clothespin | D/W/U |
| P6 | 74 | White | Non-Hispanic | Female | ID | Snack | Dice Roll | D/W/U |
| P7 | 53 | White | Non-Hispanic | Female | ID | Snack | Dice Roll | D/W/U |
| P8 | 58 | White | Non-Hispanic | Male | ID | Snack | Clothespin | D/W/U |
| P9 | 60 | White | Non-Hispanic | Female | ID | Sound | Dice Roll | Home |
| P10 | 78 | White | Non-Hispanic | Male | ID; OI | Snack | Dice Roll | Home |
| P11 | 70 | White | Non-Hispanic | Male | ID; VI | Sound | Dice Roll | Home |
| P12 | 58 | White | Non-Hispanic | Female | ID | Snack | Dice Roll | Home |
| P13 | 30 | White | Non-Hispanic | Female | ID | Snack | Dice Roll | Home |
| P14 | 62 | White | Non-Hispanic | Female | ID | Snack | Dice Roll | Home |
| P15 | 18 | White | Non-Hispanic | Female | ID | Drink | Dice Roll | Home |
| P16 | 43 | White | Non-Hispanic | Male | ID; ASD | Snack | Dice Roll | Home |
| P17 | 48 | Black | Non-Hispanic | Male | ID | Snack | Dice Roll | Home |
| P18 | 36 | White | Non-Hispanic | Male | ID; ASD | Snack | Dice Roll | Home |
| P19 | 74 | White | Non-Hispanic | Female | ID | Snack | Dice Roll | Home |
| P22 | 20 | White | Non-Hispanic | Female | ID; SLI | Snack | Dice Roll | Home |
| P23 | 21 | White | Hispanic | Male | ID; ASD; SLI | Snack | Dice Roll | D/W/U |
| P24 | 19 | Black | Non-Hispanic | Female | ID | Snack | Dice Roll | D/W/U |
| P28 | 34 | White | Non-Hispanic | Male | OI | Snack | Dice Roll | D/W/U |
| P29 | 31 | White | Non-Hispanic | Male | ID | Snack | Dice Roll | D/W/U |
| P30-B | 22 | Asian (Indian) | Non-Hispanic | Male | ASD | Snack | Dice Roll | D/W/U |
| P31 | 60 | White | Non-Hispanic | Female | ID | Other | Dice Roll | Home |
| P32 | 70 | White | Non-Hispanic | Female | ID | Other | Dice Roll | Home |
| P33 | 57 | White | Non-Hispanic | Female | ID | Other | Dice Roll | Home |
| P34 | 69 | White | Non-Hispanic | Female | ID | Other | Dice Roll | Home |
| P36 | 32 | Black | Non-Hispanic | Male | ID | Sound | Dice Roll | D/W/U |
| P38 | 70 | White | Non-Hispanic | Trans-Male | ID | Snack | Dice Roll | D/W/U |
| P41 | 19 | White | Non-Hispanic | Female | ID | Snack | Dice Roll | D/W/U |
| P43 | 22 | White | Non-Hispanic | Female | ID | Snack | Dice Roll | D/W/U |
| P44 | 42 | Asian (Indian) | Non-Hispanic | Male | ID | Snack | Dice Roll | D/W/U |
| P45 | 33 | White | Non-Hispanic | Male | ID; ASD | Snack | Dice Roll | Home |
| P46 | 24 | Black | Non-Hispanic | Male | ID; OHI | Snack | Dice Roll | D/W/U |
| P47 | 53 | White | Non-Hispanic | Male | OHI | Snack | Dice Roll | D/W/U |
| P48 | 48 | White | Non-Hispanic | Male | ID; ASD | Snack | Dice Roll | D/W/U |
| P49 | 27 | White | Non-Hispanic | Trans- Female | ID | Snack | Dice Roll | D/W/U |
| P50 | 27 | White | Non-Hispanic | Male | ASD | Snack | Dice Roll | D/W/U |
| P51 | 38 | White | Non-Hispanic | Male | ID | Sound | Dice Roll | D/W/U |
| P52-B | 22 | White | Non-Hispanic | Male | ID | Snack | Dice Roll | D/W/U |
| P53 | 31 | White | Non-Hispanic | Male | ID; OHI | Snack | Dice Roll | D/W/U |
| P54 | 56 | White | Non-Hispanic | Male | ID; OHI | Snack | Dice Roll | D/W/U |
| P55 | 47 | White | Non-Hispanic | Male | TBI | Snack | Dice Roll | D/W/U |
| P56 | 57 | White | Non-Hispanic | Male | OHI | Snack | Dice Roll | D/W/U |
| P57 | 33 | White | Non-Hispanic | Male | ID | Snack | Dice Roll | D/W/U |
| P58 | 34 | White | Non-Hispanic | Male | ID | Sound | Dice Roll | D/W/U |
| P59-B | 29 | White | Non-Hispanic | Female | ID; ASD; OHI | Snack | Dice Roll | D/W/U |
| P60-B | 34 | White | Non-Hispanic | Male | ID | Snack | Dice Roll | D/W/U |
| P61 | 22 | White | Non-Hispanic | Male | ID | Snack | Dice Roll | Home |
| P62-B | 29 | White | Non-Hispanic | Male | ID | Snack | Dice Roll | D/W/U |
| P63 | 51 | White | Non-Hispanic | Male | ID | Snack | Dice Roll | D/W/U |
| P65 | 38 | White | Non-Hispanic | Male | ID | Sound | Dice Roll | D/W/U |
| P66 | 39 | White | Non-Hispanic | Female | ID | Drink | Dice Roll | Home |
| P67 | 58 | White | Non-Hispanic | Male | ID | Drink | Dice Roll | D/W/U |
| P68 | 54 | White | Non-Hispanic | Female | ID | Drink | Dice Roll | Home |
| P69 | 61 | White | Non-Hispanic | Male | ID; ASD | Sound | Dice Roll | D/W/U |
| P70 | 20 | American Indian/Alaska Native | Non-Hispanic | Female | OHI | Snack | Dice Roll | Home |
| P71 | 34 | White | Non-Hispanic | Male | OHI | Snack | Dice Roll | D/W/U |
| P72 | 46 | White | Non-Hispanic | Female | ID | Snack | Dice Roll | D/W/U |
| P73 | 31 | White | Non-Hispanic | Female | OHI | Snack | Dice Roll | Home |
| P74 | 26 | White | Non-Hispanic | Female | ID | Snack | Dice Roll | D/W/U |
| P75 | 25 | White | Non-Hispanic | Male | ID; ASD; OHI | Snack | Dice Roll | Home |
| P76 | 32 | White | Non-Hispanic | Female | ID | Drink | Dice Roll | D/W/U |
| P77 | 55 | White | Non-Hispanic | Male | ID | Snack | Dice Roll | D/W/U |
| P78 | 41 | White | Non-Hispanic | Female | TBI | Snack | Dice Roll | D/W/U |
| P79 | 39 | White | Non-Hispanic | Male | ID; ASD | Snack | Dice Roll | D/W/U |
| P80 | 39 | White | Non-Hispanic | Male | ID | Drink | Dice Roll | D/W/U |
| P81 | 35 | White | Non-Hispanic | Male | ID; ASD | Snack | Dice Roll | D/W/U |
| P82 | 41 | White | Non-Hispanic | Male | ID | Snack | Dice Roll | D/W/U |
| P83 | 34 | White | Non-Hispanic | Male | ID | Snack | Dice Roll | D/W/U |
| P85 | 27 | White | Non-Hispanic | Female | ID | Snack | Dice Roll | D/W/U |
| P86 | 36 | White | Non-Hispanic | Male | ID | Video | Dice Roll | D/W/U |
| P87 | 25 | White | Non-Hispanic | Female | ID | Snack | Dice Roll | D/W/U |
| P88 | 33 | White | Non-Hispanic | Female | ID | Craft | Dice Roll | D/W/U |
| P90 | 31 | White | Non-Hispanic | Male | ID | Snack | Dice Roll | D/W/U |
| P91 | 27 | White | Non-Hispanic | Female | ID | Snack | Dice Roll | D/W/U |
| P92 | 34 | White | Non-Hispanic | Female | ID | Snack | Dice Roll | D/W/U |
| P93 | 30 | White | Non-Hispanic | Female | ID | Snack | Dice Roll | D/W/U |
| P94 | 32 | White | Non-Hispanic | Female | ID | Snack | Dice Roll | D/W/U |
| P95 | 26 | White | Non-Hispanic | Female | TBI | Snack | Dice Roll | D/W/U |
| P96 | 23 | Other | Non-Hispanic | Male | ASD | Video | Dice Roll | D/W/U |
| M1 | 61 | White | Non-Hispanic | Male | ASD | Snack | Dice Roll | D/W/U |
| M2 | 31 | White | Non-Hispanic | Male | ASD | Snack | Dice Roll | D/W/U |
| M3 | 37 | White | Non-Hispanic | Female | ID | Snack | Dice Roll | D/W/U |
| M4 | 32 | White | Non-Hispanic | Male | ID | Snack | Dice Roll | D/W/U |
| M5 | 24 | White | Non-Hispanic | Male | ASD | Snack | Dice Roll | D/W/U |
| M6 | 46 | Black | Non-Hispanic | Female | ID | Snack | Dice Roll | D/W/U |
| M7 | 35 | White | Non-Hispanic | Male | ID | Snack | Dice Roll | D/W/U |
| M8 | 25 | White | Non-Hispanic | Female | ID | Snack | Dice Roll | D/W/U |

*Note*. ID = Intellectual Disability; OHI = Other Health Impairment; OI = Orthopedic Impairment; Visual Impairment; SLI = Speech or Language Impairment; TBI = Traumatic Brain Injury; 1^0^ Appt Setting = Primary appointment setting; D/W/U = Day Program/Workshop/University.

**Supporting Information E**

*Appointment Mechanics*

**Prior to the appointment**

1. Prepare reinforcers (e.g., equate size/quantity) before going to appointment and ensure you have a sufficient amount
2. Call to let relevant stakeholders know that you are coming and confirm they can receive you 3. Drive to location
4. Say “hi” to everyone (smile and respond to comments in contextually appropriate ways)
5. Set up your materials
6. Invite relevant participant to come work with you
7. Initiate a 5-min casual conversation with participant before you begin any assessment

**During the appointment**

8. Display apparatus for the target response, a low-preferred alternative activity, and a “stop” card.
9. Paraphrase the following statement:

“We are going to start a session, now. This just means I’m going to give you an opportunity to earn (reinforcer). During sessions I cannot talk to you. That’s just a rule I have to follow. It doesn’t mean I don’t want to talk or that I’m not happy. If you want (reinforcer) after the session starts, you can work on (target response). If you don’t want (reinforcer), or would rather talk to me, you only need to stop (target response) for one minute. You can also just say, ‘I’m done’ or point to this stop card. While you wait, you can (alternative activity) if you like.”

10. Initiate relevant assessment. Document outcomes as needed.
11. When the appointment is over, calculate the number of “punches” the participant has earned, administer them, and have both you and participant initial relevant cells in payment tracker.
12. (When relevant) pay participant gift cards.

**After the appointment**

13. Clean up your area
14. Let everyone know you’re leaving and say “good bye”
15. Go somewhere with the internet (coffee shop, campus, your house).
16. Calculate IOA and PF (when relevant)
17. Update paper data summary files in participant binder (if this hasn’t already been done), update EDS on Box, transfer raw Countee data files to relevant Box folders.
18. Attend to “reserves” (e.g., number of reinforcers remaining) and document need for purchases when need is anticipated.

Debriefing Statement Following Study Completion

“That last session was a little weird, wasn’t it? Did you notice that I stopped giving you (reinforcer) when you (response)? The truth is, that wasn’t an accident. This entire study was to help me understand how much work you would do before you stopped when I stopped (reinforcer). You did great and I learned so much! Now that the study is over, you won’t have to (response) anymore, isn’t that great!

Since this was the last time you’ll be working on this project, let’s take a look at you punch card and see how much money I still owe you.”

**Supporting Information F**

*Procedural Fidelity Checklist for Free Access to Target Reinforcer*

Session:

Observer initials (prim/reli):

|  | **Procedure** | **Yes** | **No** | **N/A** |
| --- | --- | --- | --- | --- |
| 1. | Extraneous materials were removed from assessment area |  |  |  |
| 2. | Session termination card and alternative stimulus present. |  |  |  |
| 3. | Facilitator paraphrased (a) *“Which of these would you most like to work for?*” (b) presented an array of edible options and (c) allowed to select their highest preferred item. |  |  |  |
| 4. | After edible selection, facilitator paraphrased the following statement: *“Wonderful. We’ll start working for this the next time I come. Right now, I’m just going to let you have as much of it as you want. Each time you take one (point) and eat it, I’ll replace it with another. When you don’t want to eat anymore don’t take anymore. Remember, you can always choose to play with this (low-preferred alternative) or tell me that you’re done or that you’d rather talk to me than eat these.”* |  |  |  |
| 5. | A plate was available for placing a single morsel of selected edible item. |  |  |  |
| 6. | A single morsel rested on the plate at all times. Each time a morsel was consumed, it was replaced. |  |  |  |
| 7. | All morsels consumed were approximately the same size, shape, and quality |  |  |  |
| 8. | The edibles “reserve” (i.e., the container from which edibles were replaced) was controlled by the implementer and concealed to the extent to which concealment was possible. |  |  |  |
| 9. | The session terminated either after 1 min of no consumption, or after the participant indicated they wanted the session to end. |  |  |  |
|  | Total |  |  |  |
|  | Yes / (Yes + No) = |  |  |  |

**Supporting Information G**

*Procedural Fidelity Checklist for Target Response Training*

Session:

Observer initials (prim/reli):

Corrective prompt (circle one):

Manual guidance	Model	Gesture	Other

| **Procedure** | **Yes** | **No** | **N/A** |
| --- | --- | --- | --- |
| 1. Extraneous materials were removed from assessment area |  |  |  |
| 2. Session termination card and alternative stimulus present. |  |  |  |
| 3. A plate upon which edible morsels of a **non-target reinforcer** could be placed was available. |  |  |  |
| 4. Facilitator presented only one task-candidate at a time. |  |  |  |
| 5. Facilitator paraphrased, *“Show me if you can do this.”* Facilitator then modeled an action for the participant to imitate which entailed both a manipulation (e.g., open the wallet) and a restoration (e.g., close the wallet), and then waited for the participant to attempt it. |  |  |  |
| 6. Facilitator discarded difficult tasks and continued to assess easily completed tasks. |  |  |  |
| 7. For easily completed tasks, facilitator paraphrased the following statement: *“You can earn (****non-target reinforcer)*** *by doing (task). Try it.”* Upon task completion, facilitator delivers morsel of (**non-target reinforcer).** *Facilitator can modify instructional procedure as needed. Please document all modifications.* |  |  |  |
| 8. Facilitator paraphrased the following statement: *“Okay, let’s practice. Every time you want more of (****non-target reinforcer),*** *do (task) one time. I will only give you one (****non-target edible reinforcer)*** *at a time, so you have to eat the ones I give you before you’ll get another one. Let’s try.”* After instruction, present a trial during with no prompting. Upon each task completion, facilitator delivered morsel of (**non-target edible reinforcer).** |  |  |  |
| 9. Facilitator discarded both tasks and reinforcers for which independent responses did not occur. |  |  |  |
| 10. Facilitator operationally defined as the target task (below) an action for which independent responses occurred. |  |  |  |
| Total |  |  |  |
| Yes / (Yes + No) = |  |  |  |

**Operational Definition of Target Task:**

**Supporting Information H**

*Procedural Fidelity Checklist for Progressive Ratio Analysis (PRA) Control*

Session #:

Observer initials (prim/reli):

| **Procedure** | **Yes** | **No** | **N/A** |
| --- | --- | --- | --- |
| 10. Extraneous materials were removed from assessment area. |  |  |  |
| 11. Session termination card and alternative stimulus present. |  |  |  |
| 12. A plate upon which edible morsels of the **target reinforcer** could be placed was available. |  |  |  |
| 13. Facilitator paraphrased the following statement:    *“We are going to start a session, now. This just means I’m going to give you an opportunity to (task). When you* *do this (model task for participant), nothing is going to happen, so only do it if you like doing it.*    *If it’s boring, don’t do it. During sessions I cannot talk to you. That’s just a rule I have to follow. It doesn’t mean I don’t want to talk or that I’m not happy. If you don’t want to wait, or would rather talk to me, you only need to stop (target response) for one minute. You can also just say, ‘I’m done’ or point to this stop card. While you wait, you can (alternative activity) if you like.”* |  |  |  |
| 14. Each time participant responds the facilitator paraphrases the following and then restarts the session    *Remember, right now I just need to know that you know that responding will not get you anything. The way that I’ll know that is when you stop responding for one minute. While you wait you can play with (low-preferred alternative). Or, if you’re bored and don’t want to wait anymore, you can say, “all done” and we’ll just end the sessions.* |  |  |  |
| 15. Control condition continues until either participant does not respond for 1 consecutive minute, or after the participant indicated they wanted the session to end. |  |  |  |
| Total |  |  |  |
| Yes / (Yes + No) = |  |  |  |

**Supporting Information I**

*Procedural Fidelity Checklist for Progressive Ratio Analysis (PRA) Test*

Session #:

Observer initials (prim/reli):

| **Procedure** | **Yes** | **No** | **N/A** |
| --- | --- | --- | --- |
| 11. Extraneous materials were removed from assessment area |  |  |  |
| 12. Session termination card and alternative stimulus present. |  |  |  |
| 13. A plate upon which edible morsels of the **target reinforcer** could be placed was available. |  |  |  |
| 14. Facilitator paraphrased the following statement:    *“We are going to start a session, now. This just means I’m going to give you an opportunity to earn (reinforcer). At first, it will be easy to earn (reinforcer), then it will take more time.*    *During sessions I cannot talk to you. That’s just a rule I have to follow. It doesn’t mean I don’t want to talk or that I’m not happy.*    *If you want (reinforcer) after the session starts, you can work on (target response).*    *If you stop wanting (reinforcer), get too bored doing (task), or just want to talk to me, you only need to stop (target response) for one minute. You can also just say, ‘I’m done’ or point to this stop card. While you wait, you can*  *(alternative activity) if you like.”* |  |  |  |
| 15. Facilitator ignored participants during the session |  |  |  |
| *16.* Facilitator delivered reinforcers according to prescribed schedule requirement throughout the entire assessment |  |  |  |
| 17. Reinforcer morsel size, shape, and quality was approximately the same for all reinforcers delivered. |  |  |  |
| 18. The session terminated either after 1 min of no responding, or after the participant indicated they wanted the session to end. |  |  |  |
| Total |  |  |  |
| Yes / (Yes + No) = |  |  |  |

**Supporting Information J**

*Procedural Fidelity Checklist for Progressive Fixed-Ratio Analysis (PFRA) Test*

Session #:

Observer initials (prim/reli): **Schedule Requirement (e.g., FR 1, FR 4, FR 7):**

|  | **Procedure** | **Yes** | **No** | **N/A** |
| --- | --- | --- | --- | --- |
| 1. | Extraneous materials were removed from assessment area |  |  |  |
| 2. | Session termination card and alternative stimulus present. |  |  |  |
| 3. | A plate upon which edible morsels of the **target reinforcer** could be placed was available. |  |  |  |
| 4. | Facilitator modeled schedule requirement while paraphrasing the following:    *In this version of the game, you can earn ___, by rolling the dice ___ times like this.*    *You can work for as long as you want to earn ____, I’ve bought a lot of them and am happy to keep giving them to you. But it’s important that you only respond for as long as you want ____. The moment you’re full, tired of responding, bored, or just want to talk you just need to say you’re done by touching this card. I’ll pay you no matter what, so only respond for as long as you want ____. Can you show me that you know how to say you’re done?*    (if participant is nonverbal, identify valid expressions of dissent) |  |  |  |
| 5. | *As needed,* facilitator counted participant responses up through first two reinforcers, then tried to ignore participants for remainder of session. However, responded to participant bids for attention in contextually appropriate ways, with reminders that facilitator should not talk while participant is earning food but that if the participant would prefer to talk, they could end the session. Every five to 10 min, facilitator checks in and asks participants if they would like to stop or if they are still happy earning reinforcers. |  |  |  |
| 6. | Reinforcer morsel size, shape, and quality was approximately the same for all reinforcers delivered. |  |  |  |
| 7. | The session terminated either after ___ min (1 min recommended, but individualization may be appropriate) of no responding, or after the participant indicated they wanted the session to end. |  |  |  |
|  | Total |  |  |  |
|  | Yes / (Yes + No) = |  |  |  |

**Supporting Information K**

*Procedural Fidelity Checklist for Extinction Challenge (Baseline)*

Session #:

Observer initials (prim/reli):

**Group Assignment:**	Cheap AO	Cheap EO	Expensive AO	Expensive EO

**Schedule Requirement** (e.g., FR 1, FR 4, FR 7):

**Session Duration:**

**Max Reinforcers per Appointment:**

**Max Appointment Duration:**

|  | **Procedure** | **Yes** | **No** | **N/A** |
| --- | --- | --- | --- | --- |
| 1. | Extraneous materials removed from assessment area. |  |  |  |
| 2. | Session termination card and alternative stimulus present. |  |  |  |
| 3. | A plate upon which edible morsels of the **target reinforcer** could be placed was available. |  |  |  |
| 4. | *As needed* (for up to first five sessions of baseline) facilitator modeled schedule requirement while paraphrasing the following:    *In this version of the game, you can earn ___, by rolling the dice ___ times like this.*    *You can work for as long as you want to earn ____, I’ve bought a lot of them and am happy to keep giving them to you. But it’s important that you only respond for as long as you want ____. The moment you’re full, tired of responding, bored, or just want to talk you just need to say you’re done by touching this card. I’ll pay you no matter what so only respond for as long as you want ____. Can you should me that you know how to say you’re done?*    (if participant is nonverbal, identify valid expressions of dissent)    For final five sessions, facilitator simply asks, “do you remember what to do?” and only provides additional information if requested. |  |  |  |
| *5.* | Facilitator delivered reinforcers according to prescribed schedule requirement throughout the entire assessment    *As needed (for up to first five sessions of baseline),* facilitator counted participant responses up through first two reinforcers, then tried to ignore participants for remainder of session. However, responded to participant bids for attention in contextually appropriate ways, with reminders that facilitator should not talk while participant is earning food but that if the participant would prefer to talk, they could end the session. |  |  |  |
| 6. | Reinforcer morsel size, shape, and quality was approximately the same for all reinforcers delivered. |  |  |  |
| 7. | The session terminated at prescribed time |  |  |  |
| 8. | Appointment parameters (listed above) were not violated |  |  |  |
|  | Total |  |  |  |
|  | Yes / (Yes + No) = |  |  |  |

**Supporting Information L**

*Procedural Fidelity Checklist for Extinction Challenge (Extinction)*

Session #:

Observer initials (prim/reli):

**Group Assignment:**	Cheap AO	Cheap EO	Expensive AO	Expensive EO

**Schedule Requirement** (e.g., FR 1, FR 4, FR 7):

**Session Duration:**

**Max Reinforcers per Appointment:**

**Max Appointment Duration:**

|  | **Procedure** | **Yes** | **No** | **N/A** |
| --- | --- | --- | --- | --- |
| 1. | Extraneous materials were removed from assessment area. |  |  |  |
| 2. | Session termination card and alternative stimulus present. |  |  |  |
| 3. | A plate upon which edible morsels of the **target reinforcer** could be placed was available. |  |  |  |
| 4. | Facilitator paraphrased the following statement:  *“We are going to start a session, now. This just means I’m going to give you an opportunity to earn (reinforcer).*    *During sessions I cannot talk to you. That’s just a rule I have to follow. It doesn’t mean I don’t want to talk or that I’m not happy.*    *If you want (reinforcer) after the session starts, you can work on (target response).*    *If you stop wanting (reinforcer), get too bored doing (task), or just want to talk to me, you only need to stop (target response) for one minute. You can also just say, ‘I’m done’ or point to this stop card. While you wait, you can (alternative activity) if you like.”* |  |  |  |
| 5. | Facilitator completes pre-session task prior to initiating extinction    **AO.** Facilitator sets up area (e.g., target response, alt response, break card) but does not require participant to respond to consume *up to* as many units of reinforcement as the most consumed during any single assessment (check free access, PRA, and PFRA data to gather this information). Once max reinforcers are consumed, or participant has indicated they are done. Prompt them to respond until they satisfy the baseline schedule requirement one time and then deliver the reinforcement. Afterward, count in and start the session.  **EO.** Facilitator sets up area (e.g., target response, alt response, break card), prompts participant to respond until they satisfy the baseline schedule requirement one time, and then deliver the reinforcement. Afterward, count in and start the session. |  |  |  |
| 6. | Facilitator tried to ignore participants for entire session. However, responded to participant bids for attention in contextually appropriate ways, with reminders that facilitator should not talk while participant is earning food but that if the participant would prefer to talk, they could end the session. |  |  |  |
| *7.* | Facilitator DELIVERED NO REINFORCERS |  |  |  |
| 8. | The session terminated at prescribed time, or after participant dissent |  |  |  |
|  | Total |  |  |  |
|  | Yes / (Yes + No) = |  |  |  |

**Supporting Information M**

*Punch Card*

**Supporting Information N**

*Participant Compensation Tracking Sheet*

Participant ID: ____________________

Gift Card Preference

Amazon	Walmart	Other: __________

| Date | Assent  (“Do you want to work with me today?”) | # of Holes Punched During Appt.  (20 min = 1 punch) | Punch Card Completed  (i.e., 10 punches) Y/N | $25 Gift Card Paid after  10 punches Y/N | Experimenter  Initial | Participant Initial |
| --- | --- | --- | --- | --- | --- | --- |
|  |  |  |  |  |  |  |
|  |  |  |  |  |  |  |
|  |  |  |  |  |  |  |
|  |  |  |  |  |  |  |
|  |  |  |  |  |  |  |
|  |  |  |  |  |  |  |
|  |  |  |  |  |  |  |
|  |  |  |  |  |  |  |
|  |  |  |  |  |  |  |
|  |  |  |  |  |  |  |
|  |  |  |  |  |  |  |
|  |  |  |  |  |  |  |
|  |  |  |  |  |  |  |
|  |  |  |  |  |  |  |
|  |  |  |  |  |  |  |
|  |  |  |  |  |  |  |
|  |  |  |  |  |  |  |

**Supporting Information P**

*Results for the Models Predicting Response Rate During Extinction (preregistered analysis)*

| Variables | BP1 | | FR 1 | | NCR | |
| --- | --- | --- | --- | --- | --- | --- |
| Considered | Estimate (SE) | p-value | Estimate (SE) | p-value | Estimate (SE) | p-value |
| Intercept | 10.39 (6.32) | .10 | 9.61 (5.58) | .09 | 11.43 (5.63) | .05 |
| RRE | -0.11 (0.32) | .74 | -0.02 (0.10) | .81 | -0.07 (0.09) | .45 |
| EO (vs. AO) | -2.53 (7.95) | .75 | 2.89 (7.64) | .71 | -1.58 (8.73) | .86 |
| Elastic (vs. Inelastic) | -11.14 (8.08) | .17 | -7.74 (7.54) | .31 | -4.54 (7.61) | .55 |
| Pmax | 2.60 (9.17) | .78 | 4.74 (9.98) | .56 | 7.53 (9.13) | .41 |
| EO * Elastic | 13.16 (8.47) | .12 | 13.29 (8.90) | .14 | 17.14 (9.85) | .09 |
| RRE* EO | 0.41 (0.33) | .22 | 0.03 (0.12) | .79 | 0.12 (0.12) | .31 |
| RRE * Elastic | 0.56 (0.34) | .10 | 0.19 (0.12) | .13 | 0.08 (0.10) | .41 |
| RRE * Pmax | 0.59 (0.47) | .21 | 0.12 (0.14) | .38 | 0.08 (0.14) | .60 |

*Note*. The variable “RRE” represents the relative reinforcer efficacy variable and changes between each model based on the table header (BP1 = breakpoint, FR 1 = fixed-ratio 1, and NCR = noncontingent reinforcement [i.e., consumption free of constraint]). EO = participants who had been deprived of reinforcement prior to extinction exposure, AO = participants whose consumption had approximated equilibrium prior to extinction exposure. Pmax = unit prices that yielded the greatest net response outputs during demand-curve analysis. An asterisk indicates statistical significance at the .05 level. SE = standard error.

**Supporting Information Q**

*Results for Models Predicting Latency to Extinction (preregistered analysis)*

| Variables | | | BP1 | | FR 1 | | NCR | |
| --- | --- | --- | --- | --- | --- | --- | --- | --- |
| Considered | | Estimate (SE) | | p-value | Estimate (SE) | p-value | Estimate (SE) | p-value |
| Intercept | 114.36 (131.89) | | | .39 | 122.91 (111.45) | .27 | 113.02 (109.16) | .30 |
| RRE | -1.21 (6.69) | | | .86 | -0.72 (1.95) | .71 | -0.42 (1.68) | .81 |
| EO | 50.87 (165.98) | | | .76 | 73.60 (152.59) | .63 | 32.02 (169.21) | .85 |
| Elastic (vs. Inelastic) | -147.30 (168.68) | | | .39 | -122.17 (150.62) | .42 | -19.21 (147.51) | .90 |
| Price Pmax | 71.57 (197.16) | | | .72 | 176.25 (162.35) | .28 | 83.38 (180.37) | .65 |
| EO * Elastic | 242.00 (176.83) | | | .18 | 245.61 (177.59) | .17 | 308.22 (190.87) | .11 |
| RRE * EO | 4.12 (6.84) | | | .55 | 1.23 (2.44) | .62 | 1.70 (2.32) | .47 |
| RRE * Elastic | 9.37 (7.03) | | | .19 | 4.13 (2.44) | .09 | 1.02 (1.95) | .60 |
| RRE * Pmax | 10.99 (10.02) | | | .28 | 0.99 (2.81) | .73 | 4.37 (2.82) | .13 |

*Note*. The variable “RRE” represents the relative reinforcer efficacy variable and changes between each model based on the table header (BP1 = breakpoint, FR 1 = fixed-ratio 1, and NCR = noncontingent reinforcement [i.e., consumption free of constraint]). EO = participants who had been deprived of reinforcement prior to extinction exposure, AO = participants whose consumption had approximated equilibrium prior to extinction exposure. Pmax = unit prices that yielded the greatest net response outputs during demand-curve analysis. An asterisk indicates statistical significance at the .05 level. SE = standard error.

**Supporting Information R**

*Model Results Predicting the Response as a Proportion of Baseline, Such That Peak Response Rate is Divided by the Mean Response Rate for Each Responder (preregistered analysis)*

| Variables | BP1 | | FR 1 | | NCR | |
| --- | --- | --- | --- | --- | --- | --- |
| Considered | Estimate (SE) | p-value | Estimate (SE) | p-value | Estimate (SE) | p-value |
| Intercept | 1.03 (0.45) | .02* | 1.11 (0.38) | < . 01* | 0.85 (0.37) | .02* |
| RRE | 0.00 (0.02) | .66 | -0.01 (0.01) | .36 | 0.00 (0.01) | .92 |
| EO | 0.16 (0.56) | .78 | 0.71 (0.52) | .18 | 0.90 (0.58) | .12 |
| Elastic (vs. Inelastic) | -0.41 (0.57) | .48 | -0.74 (0.51) | .16 | -0.45 (0.50) | .37 |
| Price Pmax | 1.09 (0.65) | .10 | 0.15 (0.54) | .78 | 0.43 (0.60) | .48 |
| EO * Elastic | 0.11 (0.60) | .85 | 0.19 (0.61) | .75 | 0.11 (0.65) | .86 |
| RRE * EO | 0.03 (0.02) | 0.15 | 0.00 (0.01) | .88 | 0.00 (0.01) | .62 |
| RRE * Elastic | 0.00 (0.02) | .99 | 0.01 (0.01) | .33 | 0.00 (0.00) | .97 |
| RRE * Pmax | -0.07 (0.03) | .04* | 0.00 (0.00) | .94 | -0.01 (0.01) | .29 |

*Note*. The variable “RRE” represents the relative reinforcer efficacy variable and changes between each model based on the table header (BP1 = breakpoint, FR 1 = fixed-ratio 1, and NCR = noncontingent reinforcement [i.e., consumption free of constraint]). EO = participants who had been deprived of reinforcement prior to extinction exposure, AO = participants whose consumption had approximated equilibrium prior to extinction exposure. Pmax = unit prices that yielded the greatest net response outputs during demand-curve analysis. An asterisk indicates statistical significance at the .05 level. SE = standard error.

**Supporting Information S**

*Model Results Predicting the Anticipated Number of Reinforcers Missed Prior to Achieving an Extinction Effect (preregistered analysis)*

| Variables | BP1 | | FR 1 | | NCR | |
| --- | --- | --- | --- | --- | --- | --- |
| Considered | Estimate (SE) | p-value | Estimate (SE) | p-value | Estimate (SE) | p-value |
| Intercept | -2.11 (13.67) | .88 | 9.33 (12.02) | .44 | 9.70 (11.86) | .42 |
| RRE | 0.71 (.69) | .31 | -0.02 (0.21) | .94 | -0.02 (0.18) | .90 |
| EO | 22.07 (17.20) | .20 | 36.84 (16.46) | .03 | 42.23 (18.39) | .02* |
| Elastic (vs. Inelastic) | 9.70 (17.48) | .58 | -7.79 (16.25) | .63 | -7.61 (16.03) | .64 |
| Price Pmax | -20.90 (19.83) | .30 | -36.03 (17.19) | .04 | -43.11 (19.22) | .03* |
| EO * Elastic | -25.21 (18.33) | .17 | -25.02 (19.16) | .20 | -25.11 (20.74) | .22 |
| RRE * EO | 0.90 (0.71) | .16 | 0.08 (0.26) | .77 | -0.05 (0.25) | .85 |
| RRE * Elastic | -0.96 (.73) | .19 | 0.05 (0.26) | .84 | 0.04 (0.21) | .84 |
| RRE * Pmax | 0.18 (1.02) | .86 | 0.30 (0.30) | .32 | 0.52 (0.30) | .09 |

*Note*. The variable “RRE” represents the relative reinforcer efficacy variable and changes between each model based on the table header (BP1 = breakpoint, FR 1 = fixed-ratio 1, and NCR = noncontingent reinforcement [i.e., consumption free of constraint]). EO = participants who had been deprived of reinforcement prior to extinction exposure, AO = participants whose consumption had approximated equilibrium prior to extinction exposure. Pmax = unit prices that yielded the greatest net response outputs during demand-curve analysis. An asterisk indicates statistical significance at the .05 level. SE = standard error.

**Supporting Information T**

*Median Regression Results Predicting Rate from Conditions of the Trial and Competing RRE*

| Variables | BP1 | | FR 1 | | NCR | |
| --- | --- | --- | --- | --- | --- | --- |
| Considered | Estimate (SE) | p-value | Estimate (SE) | p-value | Estimate (SE) | p-value |
| Intercept | 01.91 (1.54) | .22 | 0.00 (1.91) | 1.00 | 4.40 (1.59) | .04* |
| RRE | 0.54 (0.12) | < .01* | 0.20 (0.06) | < .01* | 0.00 (0.03) | 1.00 |
| EO | 5.80 (2.65) | .03 | 4.40 (2.65) | .10 | 5.00 (2.96) | .09 |
| Elastic (vs. Inelastic) | -0.38 (1.92) | .84 | -0.60 (2.55) | .81 | -1.00 (2.78) | .72 |
| Price Pmax | 4.37 (5.47) | .43 | 6.80 (5.77) | .24 | 2.40 (7.32) | .74 |
| EO * Elastic | 4.18 (6.25) | .51 | 7.40 (7.03) | .30 | 12.60 (9.26) | .18 |

*Note*. The variable “RRE” represents the relative reinforcer efficacy variable and changes between each model based on the table header (BP1 = breakpoint, FR 1 = fixed-ratio 1, and NCR = noncontingent reinforcement [i.e., consumption free of constraint]). EO = participants who had been deprived of reinforcement prior to extinction exposure. AO = participants whose consumption had approximated equilibrium prior to extinction exposure. Pmax = unit prices that yielded the greatest net response outputs during demand-curve analysis. An asterisk indicates statistical significance at the .05 level. SE = standard error.

**Supporting Information U**

*Median Regression Results Predicting Latency from Conditions of the Trial and Competing RRE*

| Variables | BP1 | | FR 1 | | NCR | |
| --- | --- | --- | --- | --- | --- | --- |
| Considered | Estimate (SE) | p-value | Estimate (SE) | p-value | Estimate (SE) | p-value |
| Intercept | -42.42 (23.76) | .08 | 20.75 (31.08) | .51 | 34.32 (19.31) | .08 |
| RRE | 7.74 (2.11) | < .01* | 1.28 (0.99) | .20 | 0.64 (0.27) | .02* |
| EO | 108.68 (33.05) | < .01* | 61.95 (69.80) | .38 | 68.85 (40.84) | .10 |
| Elastic (vs. Inelastic) | 12.74 (31.41) | .69 | 6.81 (46.45) | .88 | 9.42 (36.91) | .80 |
| Price Pmax | 89.47 (63.77) | .16 | 121.95 (82.73) | .14 | 137.30 (95.28) | .15 |
| EO * Elastic | 37.89 (59.82) | .53 | 89.67 (92.13) | .33 | 99.23 (118.33) | .40 |

*Note*. The variable “RRE” represents the relative reinforcer efficacy variable and changes between each model based on the table header (BP1 = breakpoint, FR 1 = fixed-ratio 1, and NCR = noncontingent reinforcement [i.e., consumption free of constraint]). EO = participants who had been deprived of reinforcement prior to extinction exposure. AO = participants whose consumption had approximated equilibrium prior to extinction exposure. Pmax = unit prices that yielded the greatest net response outputs during demand-curve analysis. An asterisk indicates statistical significance at the .05 level. SE = standard error.

**Supporting Information V**

*Median Regression Results Predicting Proportion of Baseline from Conditions of the Trial and Competing RREs*

| Variables | BP1 | | FR 1 | | NCR | |
| --- | --- | --- | --- | --- | --- | --- |
| Considered | Estimate (SE) | p-value | Estimate (SE) | p-value | Estimate (SE) | p-value |
| Intercept | 0.63 (0.26) | .02* | 0.65 (0.25) | .01* | 0.55 (0.26) | .04* |
| RRE | 0.00 (0.01) | .98 | 0.00 (0.00) | .77 | 0.00 (0.00) | .70 |
| EO | 0.19 (0.39) | .63 | 0.20 (0.38) | .60 | 0.20 (0.37) | .59 |
| Elastic (vs. Inelastic) | -0.33 (0.29) | .25 | -0.34 (0.31) | .27 | -0.26 (0.29) | .37 |
| Price Pmax | 0.24 (0.34) | .48 | 0.24 (0.33) | .47 | 0.27 (0.33) | .41 |
| EO * Elastic | 0.65 (0.46) | .16 | 0.72 (0.45) | .12 | 0.64 (0.45) | .15 |

*Note*. The variable “RRE” represents the relative reinforcer efficacy variable and changes between each model based on the table header (BP1 = breakpoint, FR 1 = fixed-ratio 1, and NCR = noncontingent reinforcement [i.e., consumption free of constraint]). EO = participants who had been deprived of reinforcement prior to extinction exposure. AO = participants whose consumption had approximated equilibrium prior to extinction exposure. Pmax = unit prices that yielded the greatest net response outputs during demand-curve analysis. An asterisk indicates statistical significance at the .05 level. SE = standard error.

**Supporting Information W**

*Median Regression Results Predicting Reinforcers Missed from Conditions of the Trial and Competing RRE*

| Variables | BP1 | | FR 1 | | NCR | |
| --- | --- | --- | --- | --- | --- | --- |
| Considered | Estimate (SE) | p-value | Estimate (SE) | p-value | Estimate (SE) | p-value |
| Intercept | 0.08 (1.34) | .95 | 1.11 (2.85) | .70 | 2.55 (2.03) | .21 |
| RRE | 0.46 (0.17) | < .01* | 0.11 (0.05) | .03* | 0.04 (0.06) | .42 |
| EO | 14.31 (9.32) | .13 | 13.33 (8.20) | .11 | 14.48 (9.41) | .13 |
| Elastic (vs. Inelastic) | -5.15 (1.26) | < .01* | -2.22 (2.74) | .42 | -2.60 (2.05) | .21 |
| Price Pmax | -9.69 (9.93) | .33 | -8.56 (8.34) | .31 | -7.61 (9.58) | .43 |
| EO * Elastic | -8.62 (9.42) | .36 | -8.78 (8.71) | .32 | -8.28 (10.28) | .42 |

*Note*. The variable “RRE” represents the relative reinforcer efficacy variable and changes between each model based on the table header (BP1 = breakpoint, FR 1 = fixed-ratio 1, and NCR = noncontingent reinforcement [i.e., consumption free of constraint]). EO = participants who had been deprived of reinforcement prior to extinction exposure. AO = participants whose consumption had approximated equilibrium prior to extinction exposure. Pmax = unit prices that yielded the greatest net response outputs during demand-curve analysis. An asterisk indicates statistical significance at the .05 level. SE = standard error.
